# Ultrathin 2D Fe-Nanosheets Stabilized by 2D Mesoporous Silica: Synthesis and Application in Ammonia Synthesis

**DOI:** 10.1021/acsami.1c06771

**Published:** 2021-06-15

**Authors:** Hua Fan, Jan Markus Folke, Zigeng Liu, Frank Girgsdies, Robert Imlau, Holger Ruland, Saskia Heumann, Josef Granwehr, Rüdiger-A. Eichel, Robert Schlögl, Elias Frei, Xing Huang

**Affiliations:** † Department of Heterogeneous Reactions, Max Planck Institute for Chemical Energy Conversion, 45470 Mülheim an der Ruhr, Germany; ‡ Department of Inorganic Chemistry, Fritz-Haber Institute of Max Planck Society, Faradayweg 4-6, 14195 Berlin, Germany; § Fuzhou University, Wulong River North Street No.2, 350108 Fuzhou, P. R. China; ∥ Materials & Structural Analysis, Thermo Fisher Scientific, Achtseweg Noord 5, 5651 GG Eindhoven, Netherlands; ⊥ Institute of Energy and Climate Research - Fundamental Electrochemistry (IEK-9), Forschungszentrum Jülich, 52425 Jülich, Germany; # Institute of Technical and Macromolecular Chemistry, RWTH Aachen University, 52074 Aachen, Germany; ∇ Institute of Physical Chemistry, RWTH Aachen University, 52074 Aachen, Germany

**Keywords:** 2D Fe, steps/kinks, mesoporous SiO_2_, encapsulation, ammonia synthesis, electron tomography

## Abstract

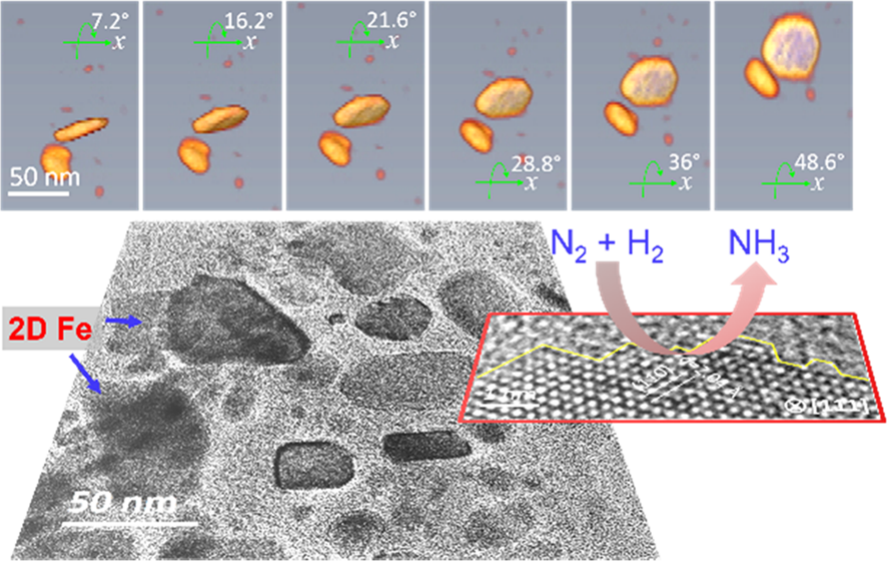

Developing high-performance Fe-based ammonia catalysts through simple and cost-efficient methods has received an increased level of attention. Herein, we report for the first time, the synthesis of two-dimensional (2D) FeOOH nanoflakes encapsulated by mesoporous SiO_2_ (mSiO_2_) via a simple solution-based method for ammonia synthesis. Due to the sticking of the mSiO_2_ coating layers and the limited spaces in between, the Fe after reduction retains the 2D morphology, showing high resistance against the sintering in the harsh Haber–Bosch process. Compared to supported Fe particles dispersed on mSiO_2_ spheres, the coated catalyst shows a significantly improved catalytic activity by 50% at 425 °C. Thermal desorption spectroscopy (TDS) reveals the existence of a higher density of reactive sites for N_2_ activation in the 2D Fe catalyst, which is possibly coupled to a larger density of surface defect sites (kinks, steps, point defects) that are generally considered as active centers in ammonia synthesis. Besides the structural impact of the coating on the 2D Fe, the electronic one is elucidated by partially substituting Si with Al in the coating, confirmed by ^29^Si and ^27^Al magic-angle spinning nuclear magnetic resonance (MAS NMR). An increased apparent activation energy (*E*_a_) of the Al-containing catalyst evidences an influence on the nature of the active site. The herein-developed stable 2D Fe nanostructures can serve as an example of a 2D material applied in catalysis, offering the chance of a rational catalyst design based on a stepwise introduction of various promoters, in the coating and on the metal, maintaining the spatial control of the active centers.

## 1 Introduction

Catalytic conversion of N_2_ and H_2_ into NH_3_ through the Haber–Bosch process is one of the most important inventions of the 20th century. Even after 100 years, we are still relying on this process to sustain an adequate food supply for the increasing world population.^1,2^ Besides its dominant use in agriculture, ammonia also serves as an important chemical for manufacturing dyes, plastics, nitric acid, etc.^1,3,4^ Recently, ammonia has even been considered as a potential hydrogen carrier due to its high hydrogen density and easy liquefaction for storage and transportation.^5−9^

The industrial catalyst for ammonia synthesis is prepared by melting Fe_3_O_4_ with different types of promoters (Al_2_O_3_, CaO, K_2_O, etc.) at ca. 2000 K.^10^ The industrial Haber–Bosch process is typically operated at a high pressure of 150–200 bar and a moderately high temperature of 400–500 °C.^11−13^ Since both, i.e., catalyst preparation and catalytic reaction, involve the use of harsh conditions, their energy consumption is extremely high. Estimation shows that about 1–2% of global energy is consumed by the industrial Haber–Bosch process each year.^11,14,15^ It is thus highly desirable to develop simple and cost-effective methods toward the synthesis of more efficient catalysts that allow operation at lower temperature for ammonia synthesis.^16,17^

Earlier studies have evidenced that ammonia synthesis is a structure-sensitive reaction.^3,18−21^ Surface sites including kinks, steps, and point defects are suggested as the active centers for dissociation of N_2_, the rate-limiting step in ammonia synthesis.^22−24^ Therefore, to gain a high activity in ammonia synthesis, developing catalysts that contain abundant surface sites is preferential. Another important concern lies in the stability of the active sites during the reaction. Drastic conditions applied in the Haber–Bosch process may result in serious sintering and loss of the active sites.^3^ Due to the complex high-temperature synthesis of the industrial catalyst, the need for an alternative synthesis approach, enabling a facile control during the catalyst generation, is required.^16,17^

Recent progress in the field of two-dimensional (2D) materials has brought unprecedented opportunities for developing novel 2D nanocatalysts for heterogeneous catalysis.^25−28^ They have emerged as important candidates for numerous reactions due to their large surface areas that potentially contain a high density of active surface sites.^29−31^ Survey of previous works, surprisingly, shows no application of 2D Fe-based catalysts yet in ammonia synthesis, although the platelet Fe has been identified already as an active phase.^32,33^ In this regard, we synthesize 2D FeOOH nanosheets encapsulated and stabilized by mesoporous SiO_2_ (mSiO_2_) and examine their performance in ammonia synthesis. The 2D Fe nanostructures, formed during an in situ activation process, are thoroughly characterized by, e.g., X-ray diffraction (XRD), N_2_ adsorption–desorption analysis, thermokinetic methods (temperature-programmed reduction (TPR) and thermal desorption spectroscopy (TDS)), and electron microscopy. Besides, the structural and electronic impact of the mSiO_2_ coating is investigated by replacing part of Si with Al. Kinetic investigations are conducted to gain information on the number and nature of the active sites. Further, the suitability of this synthesis concept for heterogeneous catalysts in general is evidenced by substituting parts of Fe by Co. A special focus is given on the catalyst structure and morphology after testing in ammonia synthesis by high-resolution transmission electron microscopy (HRTEM) and three-dimensional (3D) tomography.

## 2 Materials and Methods

### 2.1 Chemicals

All chemicals were of analytical grade and used without further treatment. FeSO_4_·7H_2_O and NaBH_4_ were purchased from Applichem GmbH (Darmstadt, Germany). Cetyltrimethylammonium bromide (CTAB) and tetraethyl orthosilicate (TEOS, 98%) were purchased from Sigma Aldrich. CoCl_2_·6H_2_O and ammonia solution (25%) were bought from VWR Prolabo. Aluminum isopropoxide (Al(OiPr)_3_) was purchased from Aldrich Chemicals.

### 2.2 Synthesis of FeOOH Nanosheets

The method for the synthesis of ultrathin FeOOH nanosheets has been reported in our previous paper published elsewhere.^34,35^ Briefly, 4.2 g of FeSO_4_·7H_2_O and 1.8 g of CTAB were dissolved in 500 mL of distilled water, which was then mixed with 20 mL (4 M) of freshly prepared NaBH_4_ solution. After the color turned black, the solution mixture was stirred in the open air for 24 h at room temperature. The product was collected by centrifugation, washed with distilled water and ethanol several times and finally dried at 60 °C.

### 2.3 Synthesis of FeOOH@mSiO_2_(CTAB)

FeOOH (1 g) of was dispersed in a mixed solution containing CTAB (5 g), distilled water (1 L), ethanol (1 L), and ammonia solution (10 mL). The obtained solution was then stirred at 40 °C for 30 min to produce a uniform dispersion. Next, 3.75 mL of TEOS was added dropwise followed by further stirring for an additional 12 h. The product was collected by centrifugation, washed with distilled water and ethanol several times and finally dried at 60 °C.

### 2.4 Synthesis of FeOOH@Al/mSiO_2_(CTAB)

Al(OiPr)_3_ was used as the Al source in the preparation. The synthesis process is similar to that of FeOOH@mSiO_2_(CTAB). The only difference is that 1 h after the addition of TEOS, Al(OiPr)_3_ (170 mg) was introduced into the solution. The nominal Si/Al atomic ratio is 20 in the initial synthetic solution.

### 2.5 Synthesis of mSiO_2_(CTAB)

The mixture of distilled water (1 L), ethanol (1 L), CTAB (5 g), and ammonia solution (10 mL) was stirred at 40 °C for 30 min. Afterward, 3.75 mL of TEOS was introduced dropwise into the solution followed by stirring for 12 h. The product was collected by centrifugation, washed with distilled water and ethanol several times and dried at 60 °C.

### 2.6 Synthesis of FeOOH/mSiO_2_(CTAB)

The Fe loading in the abovementioned coated catalyst (reduced) is estimated to be 38.7 wt %. A similar value is desired in the supported catalyst so that their performance could be fairly compared later. Therefore, 658.2 mg of FeOOH and 1.0 g of CTAB/mSiO_2_ were mixed with 500 mL of distilled water. The solution was then stirred in the open air at room temperature for 24 h. The product was collected by centrifugation, washed with ethanol and finally dried at 60 °C.

### 2.7 Synthesis of Fe_0.9_Co_0.1_hydroxide@mSiO_2_(CTAB)

The synthesis process is similar to that of FeOOH@mSiO_2_(CTAB). The only difference is that 3.75 g of FeSO_4_·7H_2_O and 356 mg of CoCl_2_·6H_2_O were used instead of 4.2 g of FeSO_4_·7H_2_O to synthesize Fe_0.9_Co_0.1_hydroxide nanosheets.

### 2.8 Characterization

The elemental analysis was performed using X-ray fluorescence (XRF) on a Bruker P4 engine. Thermogravimetric (TG) analysis was performed using a Netzsch STA 449 Jupiter thermoanalyzer. The Brunauer–Emmett–Teller (BET) surface area (*S*_BET_) was measured by a volumetric N_2_-physisorption setup (Autosorb-6B, Quantachrome) at 77 K. Powder X-ray diffraction (XRD) characterization was carried out on a Bruker D8 Advance reflection diffractometer equipped with a Lynx Eye energy discriminating position sensitive detector (1D-PSD) using Cu Kα radiation. The magic-angle spinning nuclear magnetic resonance (MAS NMR) measurements were performed on the precatalysts after calcination at 550 °C for 6 h. The calcination process was applied to remove the CTAB template and to stabilize the mesoporous (Al/)mSiO_2_ coating. In addition, the FeOOH nanosheets transform into the corresponding oxide of Fe (Fe_2_O_3_).^35^ The ^29^Si and ^27^Al NMR spectra were acquired using a Bruker 800 MHz Avance Neo spectrometer with a 3.2 mm probe at room temperature. The magic-angle spinning (MAS) rate is 20 kHz and the pulse sequence is Hahn echo. For ^29^Si NMR measurement, the 90° pulse and the recycle delay are 5 μs and 52 s, respectively. For ^27^Al NMR acquisition, the 90° pulse and the recycle delay are 2 μs and 1 s, respectively. The ^29^Si shift is referenced to octakis(trimethylsiloxy)silsesquioxane (Q8M8) (11.9 ppm) and the ^27^Al shift is referenced to 1 M Al(NO_3_)_3_ aqueous solution (0 ppm). Temperature-programmed reduction (TPR) experiments were conducted with 5% H_2_/Ar at a flow rate of 80 mL min^–1^ in a fixed-bed reactor. The samples were heated from room temperature to 700 °C^36^ at 6 °C min^–1^ with an isothermal holding period of 90 min. Scanning electron microscopy (SEM) was carried out using a Hitachi S-4800 SEM equipped with a field emission gun. Transmission electron microscopy (TEM) was carried out using an aberration-corrected JEOL ARM-200CF transmission electron microscope operated at 200 kV. Electron tomography was performed using a Thermo Fisher Scientific Talos F200X operated at 200 kV. The tomographic tilt-series were acquired by scanning transmission electron microscopy (STEM) using a high-angle annular dark-field (HAADF) detector and a Fischione 2020 tomography holder. Images were recorded every 3° in the tilt range of −64 to +68°. The images of the tilt-series were spatially aligned by a cross-correlation algorithm using Inspect 3D software, which was also used to reconstruct the 3D volume using a simultaneous iterative reconstruction technique (SIRT) algorithm. Visualization of the tilt-series and 3D volume was performed using Inspect 3D and Avizo software, respectively. Thermal desorption spectroscopy (TDS) was applied for the temperature-programmed desorption of nitrogen. Therefore, a self-constructed setup that enables the testing of powder samples was used. The setup is equipped with mass flow controllers, an IR-light furnace (Behr IRF 10), and a mass spectrometer (Pfeiffer Vacuum QME 200). The powder sample is placed on a small quartz glass boat that is placed in a quartz tube (inner diameter of 14 mm, outer diameter of 20 mm, length of 450 mm) located inside the furnace and connected to the system using Ultra Torr vacuum fittings. Afterward, the system was stepwise brought to 9 × 10^–7^ mbar and directly connected to the mass spectrometer. The reduction was carried out at 600 °C at 1 bar for 30 h (for FeOOH nanosheets, it was 500 °C for about 9 h) in a flow of 75% H_2_ in N_2_. For the nitrogen adsorption at 250 °C, the samples were reduced at 600 °C in 75% H_2_ in Ar (again for 30 h), cooled to 250 °C and treated for 1 h with 75% H_2_ in N_2_. The TDS measurements were conducted at a heating rate of 25 °C min^–1^.

### 2.9 Catalytic Testing

The catalysts were first pressed into pellets and sieved into grains with a size fraction of 250–355 μm. Afterward, 1 g of sieved catalysts (diluted with 1 g of SiC) were loaded into a fixed-bed flow reactor and activated in situ at 500 °C (1 °C min^–1^) for 14–16 h in 75% H_2_/N_2_ (440 NmL min^–1^). After completion of the reduction, the pressure was raised to 90 bar while the temperature was kept at 500 °C. The total flow rate of 75% H_2_/N_2_ was adjusted to 200 NmL min^–1^, keeping the temperature constant for 10 h. Reaction temperatures were varied between 325 and 500 °C in 25 °C steps (1 °C min^–1^). The produced NH_3_ was monitored quantitatively with an IR detector (Emerson X-stream). The apparent activation energies were calculated using the data in the low-temperature region (below 10% of the thermodynamic equilibrium).

## 3 Results and Discussion

### 3.1 Synthesis Strategy

**Figure 1:**
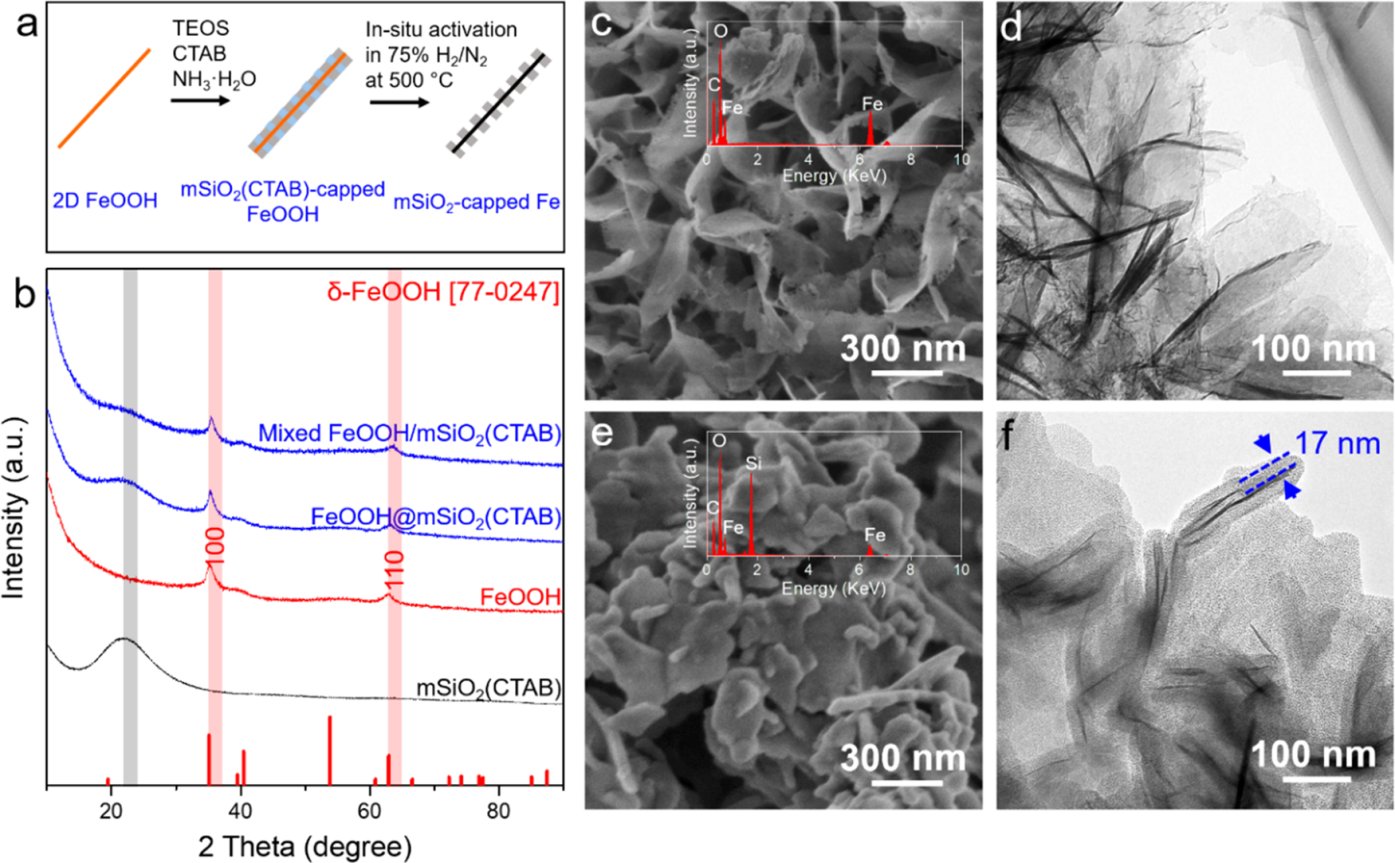
(a) Schematic representation of the fabrication process for the 2D core–shell Fe@mSiO_2_ catalyst; (b) XRD patterns of mSiO_2_(CTAB) spheres and the as-prepared FeOOH nanosheet-based precatalysts; and (c, e) SEM and (d, f) TEM images of FeOOH nanosheets and mSiO_2_(CTAB)-capped FeOOH, respectively.

The strategy toward the synthesis of a mSiO_2_-capped 2D Fe catalyst is illustrated in Figure 1a. The starting materials are FeOOH nanosheets, which were synthesized via a solution-based method, as reported in our previous study.^34,35^ In the following, we dressed FeOOH nanosheets with a layer of mSiO_2_(CTAB) composites via the Stöber-solution growth approach,^37,38^ forming a layered core–shell structure (Figure 1a). Note, CTAB was applied as a soft template for mesopore generation in the SiO_2_ layer. In situ activation (75% H_2_/N_2_, 500 °C) would eliminate CTAB completely and lead to the formation of mesopores in SiO_2_. This process would also give rise to the reduction of FeOOH to metallic Fe. In addition, to discriminate between the morphology of the active Fe moieties and the role of the coating (influence of the metal–support interaction), mSiO_2_(CTAB) sphere-supported FeOOH nanosheets and an Al/mSiO_2_(CTAB)-coated FeOOH sample (part of Si substituted by Al) were prepared.

### 3.2 Characterization of the Precatalysts

The composition of the as-prepared samples is analyzed quantitatively by the combination of X-ray fluorescence (XRF) and thermogravimetric (TG) analysis (see Figure S1). The results are listed in Table 1. The loading amount of Fe in the coated sample is measured as 24.3 wt % while a similar value (25.5 wt %) is determined for the supported one. Since both samples show a SiO_2_/CTAB mass ratio of 1.7, one can expect that after removal of CTAB during the activation process, the total Fe loading should be close (ca. 38.7 and 40.4% for coated and supported samples, respectively). The sample with an Al dopant contains slightly less Fe (21.9 wt %) in the precursor state and after activation, it is expected to be ca. 34.6 wt %. The corresponding Si/Al atomic ratio is 20.6, consistent with its nominal value.

**Table 1:** Physical Properties of the As-Prepared Catalysts

	Fe loading (wt %)			
	catalyst	precursor	SiO_2_:CTAB mass ratio	*S*_BET_ (m^2^ g^–1^)
mSiO_2_(CTAB)			1.9	18.7
mSiO_2_				900.5
FeOOH nanosheets	100	62.9		151.6
FeOOH@mSiO_2_(CTAB)	38.7	24.3	1.7	80.1
FeOOH@Al/mSiO_2_(CTAB)	34.6	21.9	1.7	63.6
mixed FeOOH/SiO_2_(CTAB)	40.4	25.5	1.7	116.6

The pore structures of samples were characterized by N_2_ adsorption–desorption analysis (Table 1 and Figure S2). As displayed in Table 1, the surface areas of mSiO_2_(CTAB) and FeOOH nanosheets are 18.7 and 151.6 m^2^ g^–1^, respectively. After the introduction of mSiO_2_(CTAB) (or Al/mSiO_2_(CTAB)) as either the coating or support materials, the derived samples show smaller surface areas (80.1 m^2^ g^–1^ for FeOOH@mSiO_2_(CTAB), 116.6 m^2^ g^–1^ for mixed FeOOH/SiO_2_(CTAB), and 63.3 m^2^ g^–1^ for FeOOH@Al/mSiO_2_(CTAB)) compared to FeOOH nanosheets. This is due to the fact that mesopores of mSiO_2_ are still blocked by CTAB. After calcination, removal of CTAB leads to a dramatically increased surface area of mSiO_2_ from 18.7 to 900.5 m^2^ g^–1^ with an average pore size of ca. 2 nm (Figure S2b). The porous structure of mSiO_2_ is stable under the harsh Haber–Bosch process, which is verified by the still remaining large surface area (800 m^2^ g^–1^) and narrow pore size (ca. 4 nm) of the spent catalyst.

Electron microscopy and X-ray diffraction (XRD) were further employed to characterize the as-prepared precursors. The scanning electron microscopy (SEM) image in Figure 1c clearly reveals that the FeOOH displays a flexible and mildly curved 2D structure. The semi-transparency feature characterized by bright-field TEM (BF-TEM) suggests that the FeOOH nanosheets are extremely thin, and according to the HRTEM image shown in Figure S3, the FeOOH sheet thickness is about 2–4 nm. Structural analysis based on the XRD pattern further reveals that the obtained FeOOH nanosheets crystallize as the δ-FeOOH phase (ICDD PDF-2 77-0247). The reflections located at 35 and 63° (2θ) can be assigned to the (100) and (110) planes of δ-FeOOH, respectively (Figure 1b).^39^ The absence of the characteristic diffraction at about 53° (2θ) can be explained by the ultrathin thickness and preferential orientation of the prepared 2D FeOOH.^34,35^

After the coating process, the FeOOH@mSiO_2_(CTAB) shows an increased thickness, as evidenced in Figure 1e. The energy-dispersive X-ray spectroscopy (EDX) spectrum shown in the inset of Figure 1e reveals a significant presence of the Si signal (compared to Figure 1c). This confirms the successful coating of the SiO_2_ layer on FeOOH nanosheets. The XRD pattern of the coated sample shows an additional peak at 22°, which can be due to the presence of amorphous SiO_2_.^40^ The ^29^Si MAS NMR spectrum of the calcined precatalyst Fe_2_O_3_@mSiO_2_ in Figure S4 indicates the dominant presence of two different coordination environments for the Si species. The peaks at chemical shifts of −110.9 and −100.0 ppm are assigned to Si sites in Si(OSi)_4_ (Q^4^, 55.3 ± 0.6%) and to Si(OSi)_3_-OH (Q^3^, 44.6 ± 0.6%), respectively.^41^ The Q^4^ Si species is located in the interior part of the SiO_2_ coating, while the latter one may be dominantly on the coating surface.^41^ In good agreement with the high surface area of mSiO_2_, the amount of the surface Si species is nearly as high as half of the total Si species. To study the thickness of the coating layer, TEM is further performed. As shown in Figure 1f, the cross-sectional view of nanosheets reveals about 17 nm of the SiO_2_ layers. As the FeOOH nanosheets are fully covered by SiO_2_ layers and the spaces between layers are constrained, we expect the 2D structured Fe to form during the activation process. Since the activation is done in situ in the reactor, and after the activation process, the catalyst is tested directly under ammonia synthesis conditions, and the activated sample is not available for characterizations. Nevertheless, the formation of mesopores in SiO_2_ is evidenced for the spent catalyst, suggesting that the activation process can efficiently remove the CTAB from pores of SiO_2_.

The XRD measurement of the supported sample (Figure 1b) shows reflections from both FeOOH and SiO_2_, which are very similar to that of the coated precursor. The mSiO_2_(CTAB) spheres alone (Figure S5a) show relatively smooth surfaces with a diameter mostly in the range from 650 nm to 1 μm. After mixing with FeOOH, the surface of the spheres becomes rather rough, considerably due to the coverage by FeOOH sheets (Figure S5b). The elemental maps of the coated and supported samples are shown in Figure S6. One can see that the distribution of Fe and Si in the coated sample is apparently more homogeneous than that in the supported one. TEM images of the supported sample (Figure S5c,d) indicate that FeOOH nanosheets and SiO_2_ spheres are not uniformly mixed.

The introduction of the Al dopant causes no obvious change in the XRD pattern of the coated sample (Figure S7). EDX measurement (Figure S8) confirms the presence of Al as part of the coating, which is distributed homogeneously. To investigate the coordination environment of Al within the coating, ^29^Si and ^27^Al MAS NMR investigations are performed on the calcined precatalyst Fe_2_O_3_@Al/mSiO_2_ (Figure S9). The signals at 9.8 and 58.4 ppm in the ^27^Al spectrum are assigned to the six-coordinated and four-coordinated Al species, respectively.^42^ This result evidenced that a significant amount of Al is successfully incorporated into the SiO_2_ framework. Comparing the Q^3^-to-Q^4^ ratio of the ^29^Si spectrum from the samples without and with Al indicates that Al–O–Si bonds are formed at the expense of Q^4^ sites.^42^ This guarantees a direct attachment of Al to the mSiO_2_ layer as part of the oxide coating and might enable investigations on the role of metal–support interactions. More discussion can be found in the Supporting Information.

**Figure 2:**
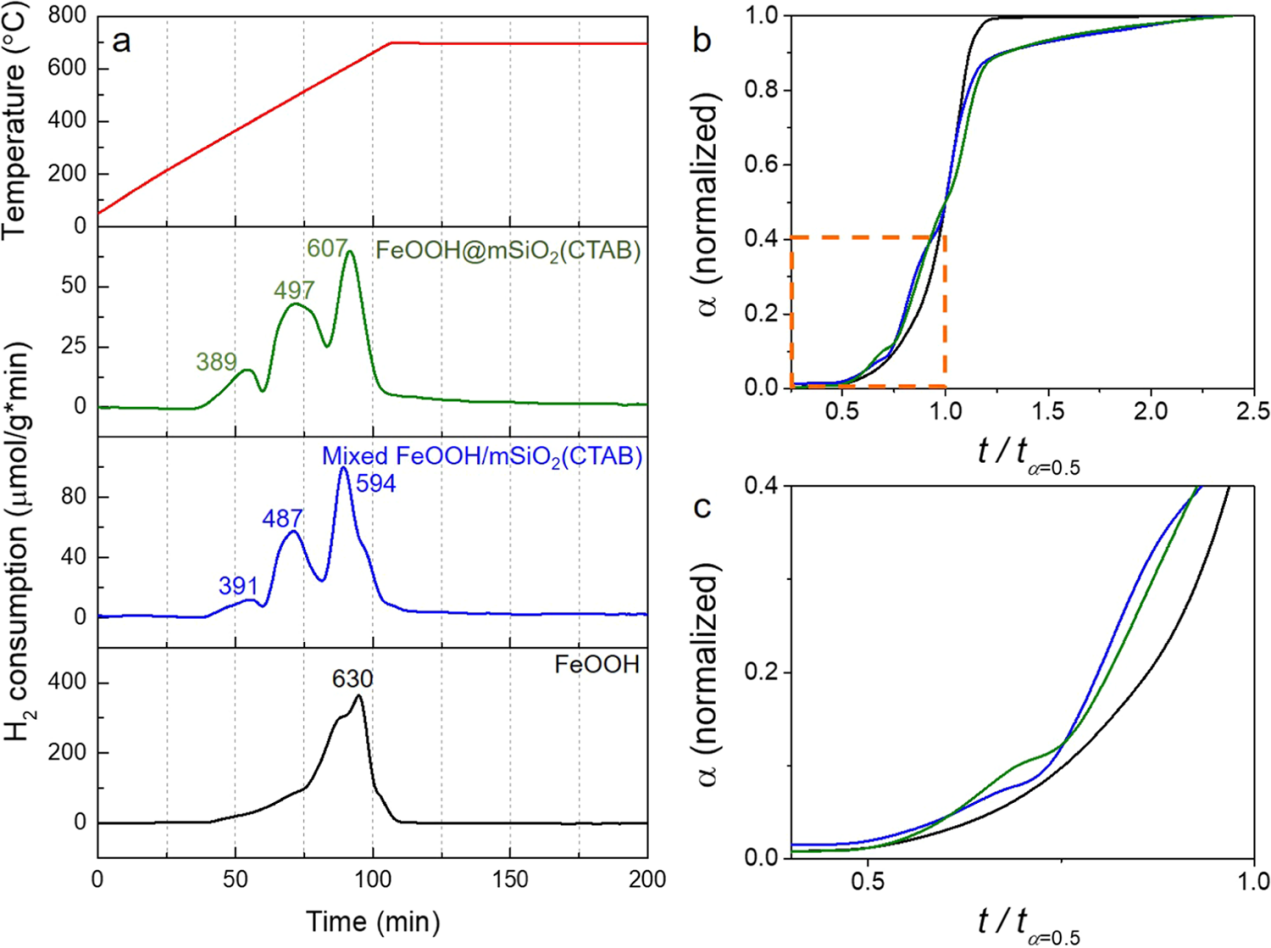
(a) H_2_-TPR of FeOOH (black), mSiO_2_-supported (blue) or -coated (green) FeOOH; (b) integrated TPR curves to time-fractions (*t*/*t*_α=0.5_); and (c) zoom-in of the selected area indicated in (b). Conditions: 5% H_2_/Ar, 80 mL min^–1^, 6 °C min^–1^, 700 °C, and 90 min holding time.

To investigate the reduction behaviors of the catalyst precursors, temperature-programmed reduction (TPR) analysis was carried out by ramping the temperature to 700 °C at a heating rate of 6 °C min^–1^ in 5% H_2_/Ar. The Al-containing sample shows an almost identical reduction behavior with the SiO_2_-coated sample and therefore is not separately discussed (see Figure S10). One can see that both, the FeOOH@mSiO_2_(CTAB) and the mixed FeOOH/mSiO_2_(CTAB), show a similar reduction behavior with the presence of three main peaks located at around 390, 490, and 600 °C (see Figure 2a). Since almost no H_2_ consumption is observed for the mSiO_2_(CTAB) (see Figure S11), the consumption peaks observed from both precursors can be solely attributed to the reduction of FeOOH. Additionally, the occurrence of reduction in the coated sample implies that the CTAB in pores of SiO_2_ layers can be fully removed during the reduction process so that it will not inhibit the reduction of the coated FeOOH. Generally, FeOOH follows a three-step reduction at elevated temperature following the path: FeOOH → Fe_3_O_4_ → FeO → Fe.^35,43^ The presented three consumption peaks respond to the reduction processes from FeOOH → Fe_3_O_4_, Fe_3_O_4_ → FeO, and FeO → Fe, which was recently shown for another FeOOH nanosheet-based system.^35^ However, for unsupported FeOOH nanosheets, the different steps are not well resolved. The maximum H_2_ consumption appears at 630 °C, which is ca. 30 °C higher compared to that for the SiO_2_-containing catalysts. The shift to a higher reduction temperature is considerably attributed to the aggregation of the nanosheets due to the lack of support materials. It needs to be mentioned that the calculated H_2_ consumption amount is still much less than the total amount needed for the full reduction, suggesting that the partial samples are not fully reduced under the applied conditions. The reduction degrees for FeOOH, FeOOH@mSiO_2_(CTAB), FeOOH/mSiO_2_(CTAB), and FeOOH@Al/mSiO_2_(CTAB) are 58, 37, 37, and 38%, respectively. To gain more insights on the reduction mechanism, independent of a temperature shift, the integrated TPR profiles are normalized to time-fractions (*t*/*t*_α=0.5_), as shown in Figure 2b,c as α-plots. One can see that the α-plots of encapsulated FeOOH and supported FeOOH show close shape and inclination, indicating a similar reduction mechanism. There are still some minor differences existing, for example, the shift in the shoulder position (*t*/*t*_α=0.5_ = 0.62–0.75), which could be due to the slightly different contact area between the metal and SiO_2_. The coated sample, in comparison to the supported one, shows a faster reduction of the first event and a slower and lasting reduction of the following steps. In contrast, the α-plot of unsupported FeOOH nanosheets shows a sharper inclination and shifts toward higher *t*/*t*_α=0.5_, suggesting a different reduction mechanism. Beyond 0.5 of the integrated area, the α-plots give information on the autocatalytic character of the reduction process. The pure Fe sample curve increases strongly, indicative of a support and dispersion-free reduction with a strong autocatalytic contribution. This strong autocatalytic character of the reduction process beyond 0.5 *t*/*t*_α_ values explains the higher degree of reduction for FeOOH (58%). The SiO_2_-containing samples show a small autocatalytic contribution due to the high dispersion.

In summary, the set of samples (supported, coated, coated with the Al dopant), investigated with respect to their structural and physicochemical properties, show only small variations attributed to the intended changes and are perfectly suitable for the analysis of their catalytic performances.

### 3.3 Catalytic Testing

In situ catalytic activation was performed on the precursors in 75% H_2_/N_2_ at 3–4 bar at a heating rate of 1 °C min^–1^ up to 500 °C. The MS data shows the formation of water prior to the onset of ammonia in SiO_2_-coated (with and without Al) and supported catalysts (Figure S12b–d), whereas for unsupported FeOOH, there is no ammonia detected (Figure S12a). The water signal generated at around 100 °C is mainly from the surface adsorbed water and that which appeared at high temperatures is due to the reduction of catalysts. In comparison with the TPR experiments that show distinguished water peaks due to different steps of reduction, serious overlapping of water peaks is observed on samples during the activation process, which is considered due to the much slower heating rate of the activation. It is also noted that the coated and supported samples show the cessation of water formation in a longer time (Δ*t* ≈ 2.5 h) than the unsupported sample, indicating a delayed reduction. This result indeed agrees well with the TPR measurements. The integrated water signal detected in all samples is larger than the amount generated for the full reduction of catalysts (see insets of Figure S12a–d). This suggests complete conversion to the metallic Fe during the activation process, particularly for the coated and supported catalysts showing already the steady-state generation of ammonia. Further, the importance of a complete reduction is shown in situ, since NH_3_ formation starts with the removal of the water signal. The extra water signal is due to the surface adsorbed water that desorbs at elevated temperatures.

**Figure 3:**
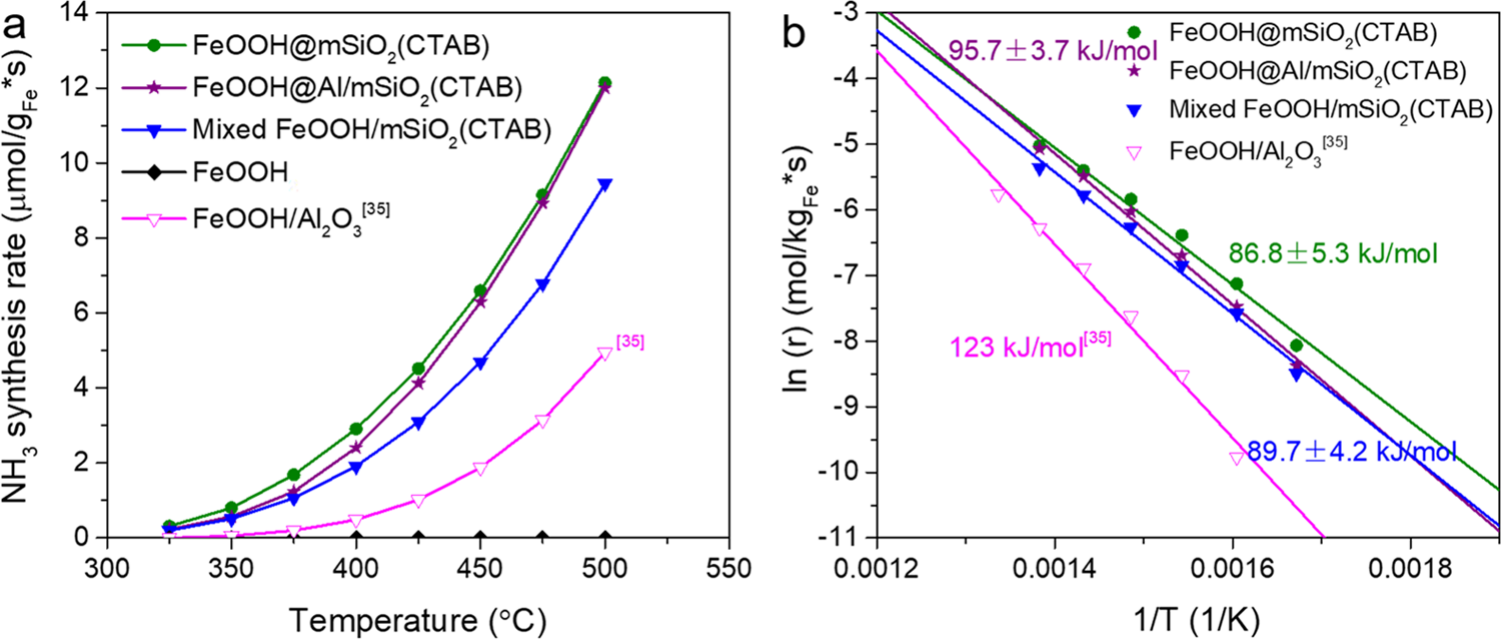
(a) Fe mass normalized NH_3_ production rates. (b) Arrhenius plots for NH_3_ synthesis at 90 bar over the γ-Al_2_O_3_-supported^35^ (pink), mSiO_2_-supported (blue), and -coated (without Al: green, with Al: purple) catalysts. Reaction conditions: 75% H_2_/N_2_, 200 NmL min^–1^, and 90 bar.

In preparation for the subsequent activity test, the pressure was then elevated to 90 bar and kept for 10 h at 500 °C. It is found that during this period the SiO_2_-supported sample shows a significant deactivation before getting stabilized (Figure S13b). However, a further increased activity is observed for the coated sample (Figure S13a,c). We propose that the reduced activity in the supported sample might be due to an increased degree of sintering induced by the pressure increase, in particular *p*(H_2_), that leads to the loss of active surfaces. Furthermore, the reaction temperature is varied stepwise from 500 to 325 °C with a 25 °C interval and back again to 500 °C. We find that the SiO_2_-coated samples are more active than the SiO_2_-supported one in the steady-state (see Figure S13). At 425 °C, the NH_3_ production rate of the SiO_2_-coated FeOOH sample amounts to 4.5 μmol g_Fe_^–1^ s^–1^, which is ≈50% more active than that of the SiO_2_-supported one (3.1 μmol g_Fe_^–1^ s^–1^) (see Figure 3a). This catalytic performance is superior to that of many other catalysts reported in the literature (see Table 2).^35,44−50^ Note, it is still inferior to that of an industrial catalyst because it does not involve any promoters yet. However, we want to emphasize here that this work focuses more on the demonstration of the design and synthesis of the active 2D Fe catalyst for ammonia synthesis. The study of promoted catalysts aiming for higher catalytic performance will be summarized in a separated work. Interestingly, despite the fact that the reaction rate of the coated and supported catalysts is different, the apparent activation energy is very close in both cases (Figure 3b). This result implies that the two catalysts with the same support material contain the same type of active species (and likely a similar metal–support interaction). It is suggested that the activity difference is defined by the number of active sites (higher dispersion), in line with the α-plot profile. When Al is incorporated into the coated catalyst, the reaction rate is slightly decreased to 4.1 μmol g_Fe_^–1^ s^–1^. Correspondingly, the apparent activation energy is elevated from 86.8 to 95.7 kJ mol^–1^ (ca. 10% higher), indicating an influence on the nature of the active sites. In general, SiO_2_ is regarded as one of the most used inert supports for numerous catalysts^51,52^ and a standard structural promoter for industrial ammonia synthesis catalysts.^3^ However, in the current catalysts, it seems that the mSiO_2_ coating is more than a structural stabilizer and electronic effects likely occur. In other words, the different interaction of Fe and the corresponding support materials (mSiO_2_ and Al/mSiO_2_) might lead to another interfacial (metal-oxide) contact and consequently to a change of the local Fe structure, and correspondingly, the active site. These findings agree well with our previous research results, where the catalytic performances of γ-Al_2_O_3_-supported FeOOH nanosheet catalysts are explained by the density of kinks and steps within the active Fe structures (see also Figure 3, FeOOH/Al_2_O_3_ performance as the reference).^35^ Obviously, SiO_2_, either as a support or a coating material, is a better support than γ-Al_2_O_3_ for the FeOOH nanosheets. This might be explained by the interfacial formation of Fe-silicide^53^ structures acting as intrinsic stable structural (more defects) and electronic (different active sites) promoters. The stability of such an interaction is confirmed by isothermal testing of catalysts at 425 °C and 90 bar for more than 45 h. All of the SiO_2_ involved catalysts show long-term durability with a negligible loss of activity (Figure S14).

**Table 2:** Catalytic Performance Comparison of Different Ammonia Synthesis Catalysts

catalyst	*p* (bar)	*T* (°C)	NH_3_ synthesis rate (μmol g_cat_^–1^ h^–1^)	ref
	3–4 (activation)	500	2484	this work
FeOOH@mSiO_2_(CTAB)	90	500	10 620	
		425	3960	
		325	273.6	
FeOOH/mSiO_2_(CTAB)	90	425	2844	this work
FeOOH/Al_2_O_3_(15% Fe)	90	425	972	(35)
Fe/Acid-modified γ-Al_2_O_3_	1	320	0.115	(44)
supported Fe prepared from amorphous Fe_91_Zr_9_	9	417	72	(45)
Co_3_Mo_3_N	100	400	5357	(46)
Fe/CeO_2_(7% Fe)	1	450	1350	(47)
TiH_2_	50	400	2800	(48)
industrial catalyst	90	425	51 480	this work

catalyst	electrolyte	NH_3_ synthesis rate (μmol g_cat_^–1^ h^–1^)	ref
Fe_SA_-N–C	0.1 M KOH	440	(49)
Fe_SA_/(O–C_2_)_4_	0.1 M KOH	1900	(50)

### 3.4 Thermal Desorption of Nitrogen

**Figure 4:**
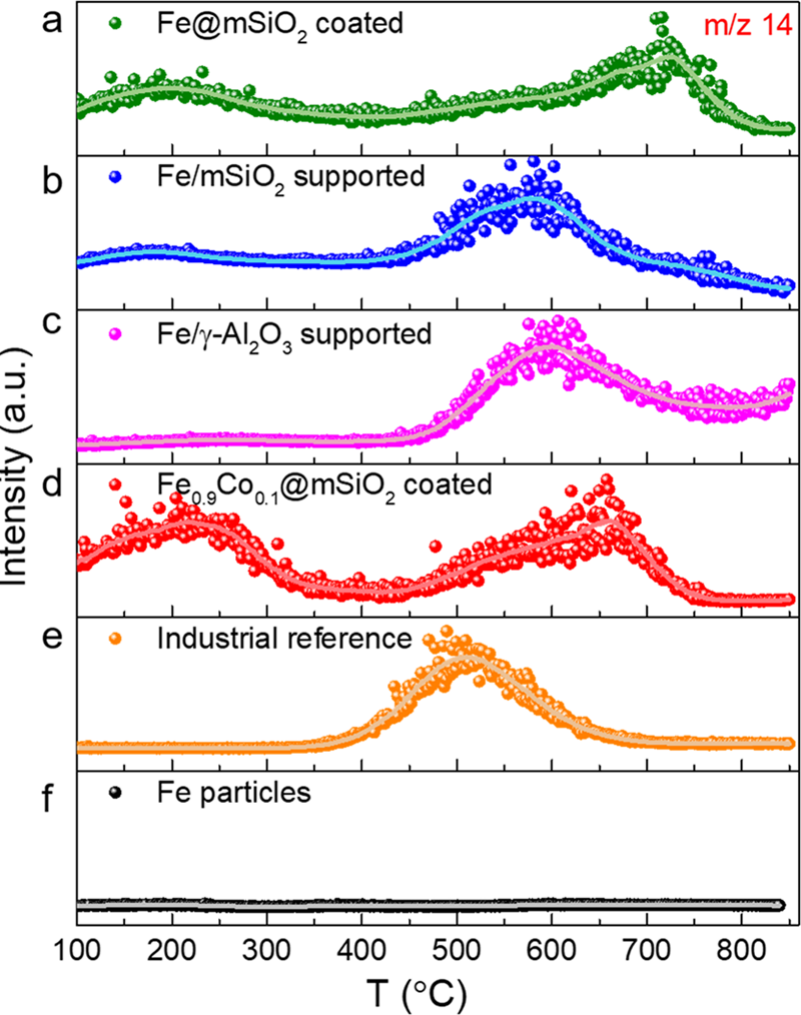
TDS spectra of (a) mSiO_2_-coated, (b) mSiO_2_-supported, (c) γ-Al_2_O_3_-supported Fe catalysts as well as (d) mSiO_2_-coated Fe_0.9_Co_0.1_, (e) industrial catalysts and (f) pure Fe particles. Most samples were activated under 1 bar in a flow of 75% H_2_/N_2_ at 600 °C for 30 h. The uncoated FeOOH nanosheets were activated under milder conditions (500 °C, 10 h) to avoid severe sintering. The TDS measurements were conducted at a heating rate of 25 °C min^–1^. The solid curves are smoothed data to guide the eye.

To access different kinds of surface sites, N_2_-TDS measurements were performed on the coated and supported Fe nanostructures. Figure 4 shows the TDS signal of the *m*/*z* = 14 with respect to the Fe mass. Prior to the desorption experiment, the samples (except for unsupported FeOOH) were activated in situ for 30 h at 600 °C under a flow of 75% H_2_/N_2_. The TDS signals differ significantly in two regions, a low-temperature desorption event at around 200 °C and a high-temperature one at 700 °C. A common event is observed at 550 °C. The low-temperature desorption is attributed to weakly bound N-species. This might be related to interfacial Fe sites (Fe–mSiO_2_ interface), highly populated in the mSiO_2_-coated system. The common event is likely attributed to defect sites such as kink and step edges, which are generally seen as active centers for the ammonia synthesis.^24^ The late desorption event of the coated sample is related to N-reconstruction of terrace sites, possibly related to the additional formation of subsurface Fe–N species. The formation of surface and subsurface nitrides is supported by a Fe_3_N reference measurement, which shows a late desorption event in the range of 650 °C (see Figure S16a). Substituting parts of the Fe by Co leads to a reduction of the high-temperature desorption, respectively, a shift to lower temperatures by ca. 80 °C. This is explained by the lower nitrogen binding energy of Co in comparison to Fe and as a consequence a lower tendency to form stable surface/subsurface reconstructions.^54^ This is also reflected in the catalytic activity of the Co-containing sample showing a higher catalytic performance and slightly lower apparent *E*_a_ (see Figure S15). Another TDS experiment shown in Figure S16b of the mSiO_2_-coated catalyst highlights the ability of the 2D nanostructures to activate N_2_ at moderate temperatures of 250 °C (reduction in 75% H_2_/Ar at 600 °C for ca. 30 h and a subsequent H_2_/N_2_ treatment at 250 °C for 1 h). The high-temperature events are significantly reduced (at 700 °C) or absent (at 550 °C). This means the terrace reconstructions and subsurface N-species are interpreted as a coverage/poisoning of the Fe surface, also formed under the harsh conditions of ammonia synthesis. This phenomenon is significantly decreased for the mSiO_2_-supported catalyst (Figure 4b). The interfacial Fe sites, responsible for the low-temperature desorption event, are, still present. As the reference experiment on the role of the support (and the interfacial Fe contact), the Al_2_O_3_-supported Fe catalysts, which have shown a lower activity and increased apparent activation energy, are analyzed with TDS (Figure 4c). The low-temperature desorption event is absent, the defect-related one is shifted by ca. 50 °C toward higher temperatures, and the reconstructions are rather pronounced. This might explain the poor catalytic performance in comparison to the SiO_2_ samples. A fully promoted industrial catalyst, still much more active than the unpromoted SiO_2_ systems, shows only one desorption event (Figure 4f). The signal likely related to defect sites (also promoter induced) is shifted by 50 °C to lower temperatures, exactly the temperature relevant for the industrially applied ammonia synthesis. The industrial reference catalyst, however, is not able to activate N_2_ at 250 °C, but the set of added promoters shifts the defects sites to lower temperatures and avoids any surface poisoning by reconstructions (no high-temperature desorption). To sum up, the 2D Fe structures stabilized by mSiO_2_ show the ability to activate N_2_ at moderate temperatures (one important criterion for high reaction rates), but on the other hand tend to lead to N-induced poisoning effects, which might be coupled to strong binding of the NH_3_ product (as another rate-determining criterion, absent in the industrial catalyst). This means, a promoter optimized 2D Fe catalyst might combine the positive effects of low-temperature N_2_ activation, low-temperature defect sites without N-reconstructions, as part of a separated study.

### 3.5 Characterization of Postreaction Catalysts

**Figure 5:**
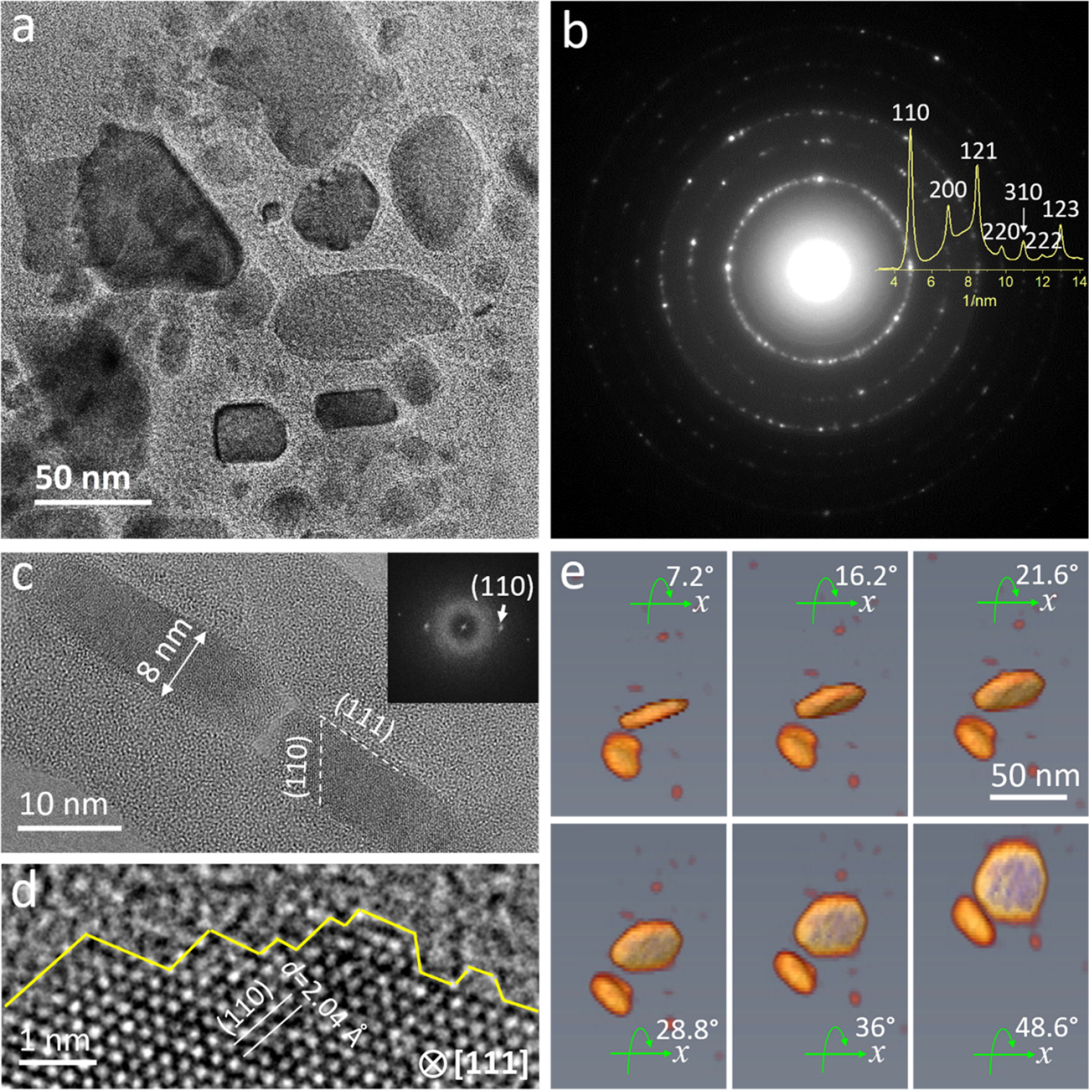
(a) TEM image and (b) selected-area electron diffraction of the spent coated catalyst; (c) HRTEM image of a cross-sectional view of the catalyst; The absence of mesopores in the SiO_2_ coating layer is due to the beam damage during high-resolution imaging; (d) HRTEM image of a plate-like particle viewed along the [111] zone axis, showing rich step edges on the surface; and (e) 3D tomography of the catalyst, confirming the formation of 2D Fe.

To gain insights into the maintained morphology, structure, and composition of the SiO_2_-coated catalyst (as a prerequisite for any optimization approaches), the spent sample was comprehensively characterized by XRD and TEM in combination with EDX and EELS techniques. Figure 5a shows a typical BF-TEM image of a Fe@mSiO_2_ core–shell structure after a series of catalytic tests. Unlike the precursor catalyst that shows a continuous sheet-like structure with a relatively weak contrast under the coating layer, the spent catalyst shows the clear presence of nanoparticles with the size ranging from a few to several tens of nanometers. Based on the secondary electron (SE) image recorded simultaneously with BF-STEM and HAADF-STEM images, shown in Figure S18, the nanoparticles are still embedded in the SiO_2_ layers. The diameter of the mesopores in the SiO_2_ layer is measured as 1.5–4 nm, as shown in Figures 5a and S19, perfectly in line with the pore distribution determined by N_2_ adsorption isotherms. To reveal the phase of the activated catalyst, selected-area electron diffraction (SAED) was taken (see Figure 5b), which assuredly demonstrates the formation of metallic Fe in a bcc structure. It is also confirmed by the XRD analysis of the spent catalyst (see Figure S21). Closer observations through the cross-sectional view further reveal that most of the nanoparticles likely have a 2D morphology with a thickness of ca. 8 nm (Figure 5c). According to the structural analysis, the top/bottom surfaces are enclosed with the (111) facet. Figures 5d and S18d show plan-view HRTEM images of nanoplates orientated along the [111] direction. The lattice fringes with *d*-spacings of 2.03–2.04 Å fit well to the (110) plane of bcc Fe. The plate-like particles show rich step edges that are commonly considered as active sites in ammonia synthesis. To further confirm the 2D structure of Fe, electron tomography, a method to construct 3D information from serial 2D images, is employed to examine the morphology of Fe. The procedure for data acquisition is provided in the Materials and Methods Section. As shown in Figure 5e, a series of images with different rotation angles along the *x*-axis clearly demonstrate the 2D morphology of Fe particles (see Movie S1). A larger view with more particles present is provided in Movies S2 and S3 (tilted STEM image series and the corresponding reconstructed 3D data. We also performed the compositional analysis using STEM-EDX (Figure S18g–j). The elemental mapping reveals the distribution of Fe that is surrounded by Si and O (Figure S18g–i). To confirm the metallic phase of Fe from an electronic structure point of view, the STEM-EELS spectrum and maps were recorded simultaneously with the EDX. The EELS map (Figure S18f) shows a similar distribution of Fe to that determined by EDX mapping (Figure S18e). Moreover, analysis of Fe L_2,3_-edges of the catalyst (Figure S18k) shows a good fit to that of metallic Fe.^55,56^

Compared to the coated catalyst, the particle dispersion in the supported spent catalyst is obviously less homogeneous (Figure S20a). Agglomeration of nanoparticles is found. Interestingly, a small fraction of particles also appear in the 2D shape, which is probably inherited from the initial 2D morphology of the FeOOH. The electron diffraction (inset of Figure S20a) evidences that the Fe particles are in the metallic phase, which is in agreement with the XRD result (see Figure S21). The HRTEM image of an [001] orientated Fe nanoparticle shows the lattice *d*-spacing of 2.04 Å, corresponding to the (110) plane of bcc Fe (Figure S20b). In contrast to the SiO_2_-coated or -supported catalysts that contain small nanoparticles, the unsupported catalyst (Figure S22) shows a much larger particle size (several tens to hundreds of nm). The particles have quite smooth surfaces dominantly terminated by the (100) and (110) planes. An increasing trend of the Fe size is also revealed by the XRD analysis (Figure S21). For the coated and supported catalysts, the domain size is estimated to be 14 nm (15 nm for the Al-containing catalyst) and 19 nm, respectively. However, for the unsupported one, the size increases profoundly to 87 nm. The serious sintering and agglomeration of catalytic particles result in the significant loss of active sites, thus presenting a negligible activity. As for the higher activity observed from the coated catalyst compared to the supported one, our experimental evidence points to the origin of the formation of 2D structures, which provides a higher density of surface steps/kinks and defective sites relevant for N_2_ dissociation and NH_3_ formation. Moreover, the combination of TEM and XRD analyses indicates a higher fraction of the active Fe(111) surface exposed in the encapsulated catalyst (detailed discussion is provided in the Supporting Information), which may also be an important reason for the superior activity compared to that of the supported one.

## 4 Conclusions

In summary, we report the fabrication of SiO_2_-encapsulated FeOOH nanosheets as a catalyst precursor for ammonia synthesis through a facile and low-cost solution-based method. Atomic-scale TEM characterization of the catalyst performed after in situ activation and a series of catalytic tests implies that the catalyst on work contains 2D Fe nanostructures (embedded into porous SiO_2_ layers) with the presence of abundant surface step/kink sites. The 2D Fe catalyst shows a higher catalytic activity compared to the supported catalyst containing Fe nanoparticles supported on the SiO_2_ spheres (1.5 times higher at 425 °C). Although the two catalysts present different activities, they show very close activation energy, indicating the same types of active species. The catalyst with Al-doped mSiO_2_ (Fe@Al/mSiO_2_) shows slightly lower activity and higher activation energy than the nondoped catalyst (Fe@mSiO_2_), suggesting that Al doping introduces a structural and electronic effect that changes the nature of the active site negatively. Substituting parts of Fe by Co leads to a more active catalyst and a positive impact on the active sites. In consideration of our experimental results based on mutual characterization techniques, we propose that the higher activity of the encapsulated catalyst is attributed to the formation of 2D Fe nanostructures that can expose more active surface sites (steps, kinks, point defects) compared to that of the supported Fe nanoparticles, highlighting the importance of shape control in catalysis. The herein-presented synthetic method could serve as an experimental basis for the rational design and economic synthesis of efficient 2D Fe-based catalysts for ammonia synthesis upon adding dedicated promoters. Moreover, this work may inspire more studies related to the preparations and applications of encapsulated 2D metal catalysts for various catalytic reactions, which may give rise to unprecedentedly high catalytic performances that are difficult to achieve from common particle catalysts.

## Supporting Information Available

Movie S1 (AVI)
Movie S2 (AVI)
Movie S3 (MP4)
XRD patterns of precursor catalysts; HRTEM image of FeOOH nanosheets; ^29^Si MAS NMR spectrum of the coated sample with and without Al; ^27^Al MAS NMR spectra of the coated sample with the Al dopant; SEM and TEM images of the mSiO_2_(CTAB) and mixed FeOOH/mSiO_2_(CTAB); SEM image and EDX mapping of coated and supported precursor catalysts; structural and compositional analyses of the coated sample with the Al dopant; TPR profiles of the coated samples with and without the Al dopant; TPR profile of mSiO_2_(CTAB); catalytic performances of different catalysts; stability tests; TDS spectra of Fe_3_N and the coated sample; XRD, TEM, and TPR of Fe_0.9_Co_0.1_hydroxide@mSiO_2_(CTAB); and morphological, structural, and compositional analyses of used catalysts (PDF)

## Supplementary Material

am1c06771_si_001.avi

am1c06771_si_002.avi
