# Supplementary material for: Ultrathin 2D Fe-Nanosheets Stabilized by 2D Mesoporous Silica: Synthesis and Application in Ammonia Synthesis
Source: ACS Appl Mater Interfaces. 2021 Jun 15;13(25):30187–97. doi: 10.1021/acsami.1c06771 (PMC8397249; doi:10.1021/acsami.1c06771)
Supplement: Supplementary file 4 — am1c06771_si_004.pdf [file am1c06771_si_004.pdf]

Supporting Information

Ultrathin 2D Fe-Nanosheets Stabilized by 2D Mesoporous Silica: Synthesis and Application in Ammonia Synthesis

Hua Fan,[a,b,c] Jan Markus Folke,[a] Zigeng Liu,[a,e] Frank Girgsdies,[b] Robert Imlau,[d] Holger Ruland,[a] Saskia Heumann,[a] Josef Granwehr,[e,f] Rüdiger-A. Eichel,[e,g] Robert Schlögl,[a,b] Elias Frei,*[b] and Xing Huang*[a,b,c]

[a]*Department of Heterogeneous Reactions, Max Planck Institute for Chemical Energy Conversion, 45470 Mülheim an der Ruhr, Germany*

[b]*Department of Inorganic Chemistry, Fritz-Haber Institute of Max Planck Society, Faradayweg 4-6, 14195 Berlin, Germany*

[c]*Fuzhou University, Wulong River North Street No.2, 350116 Fuzhou, P. R. China*

[d]*Thermo Fisher Scientific, Materials & Structural Analysis, Achtseweg Noord 5, 5651 GG Eindhoven, Netherlands*

[e]*Forschungszentrum Jülich, IEK-9, 52425 Jülich, Germany*

[f]*RWTH Aachen University, Institute of Technical and Macromolecular Chemistry, 52074 Aachen, Germany*

[g]*RWTH Aachen University, Institute of Physical Chemistry, 52074 Aachen, Germany*

Emails: eliasfrei@web.de; xinghuang@fzu.edu.cn

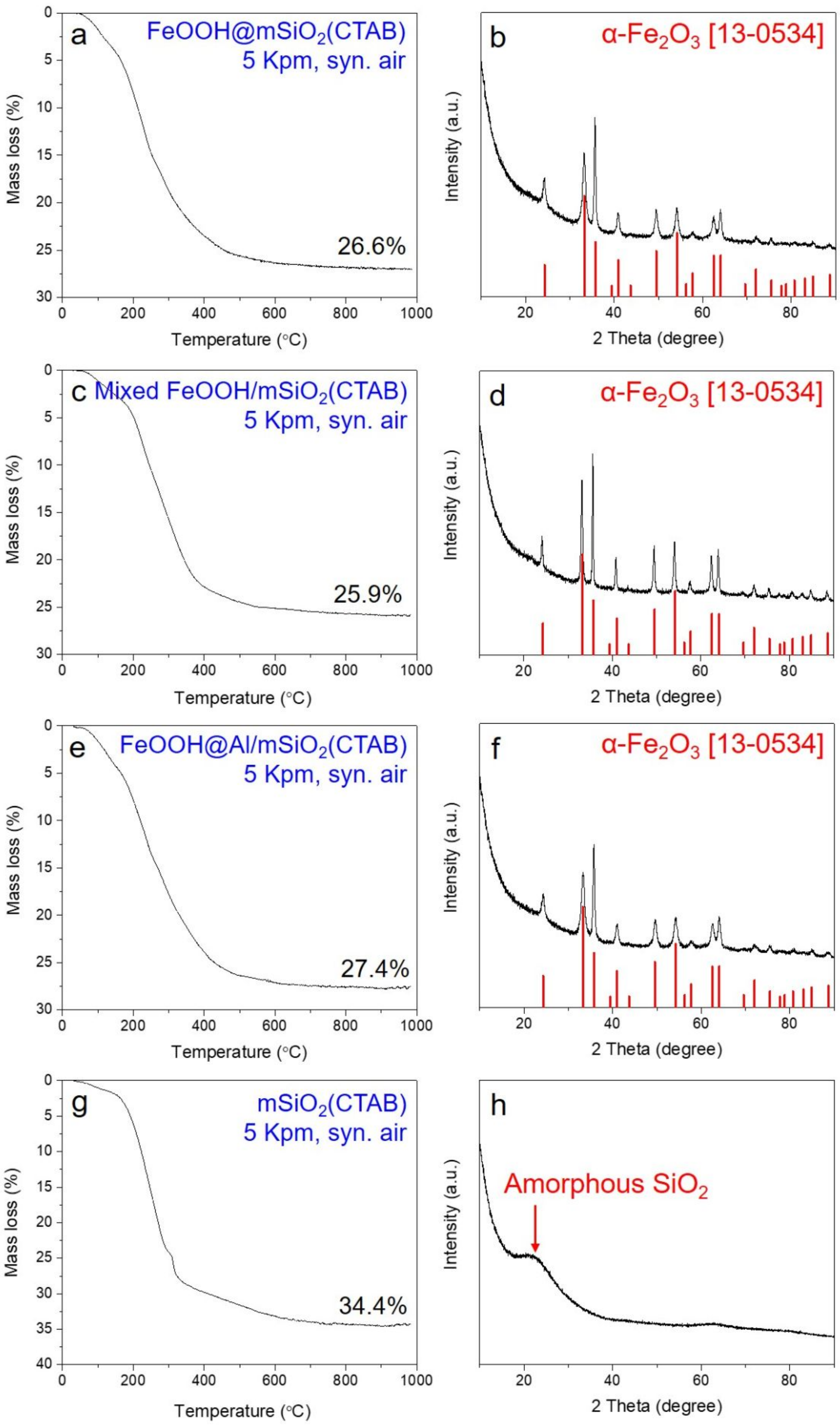

Figure S1. TG curves during calcination (in Air) and the corresponding XRD patterns after TG measurements of (a,b) FeOOH@mSiO_2(CTAB), (c,d) mixed FeOOH/mSiO_2(CTAB), (e,f) FeOOH@Al/mSiO_2(CTAB) and (g,h) mSiO_2(CTAB).

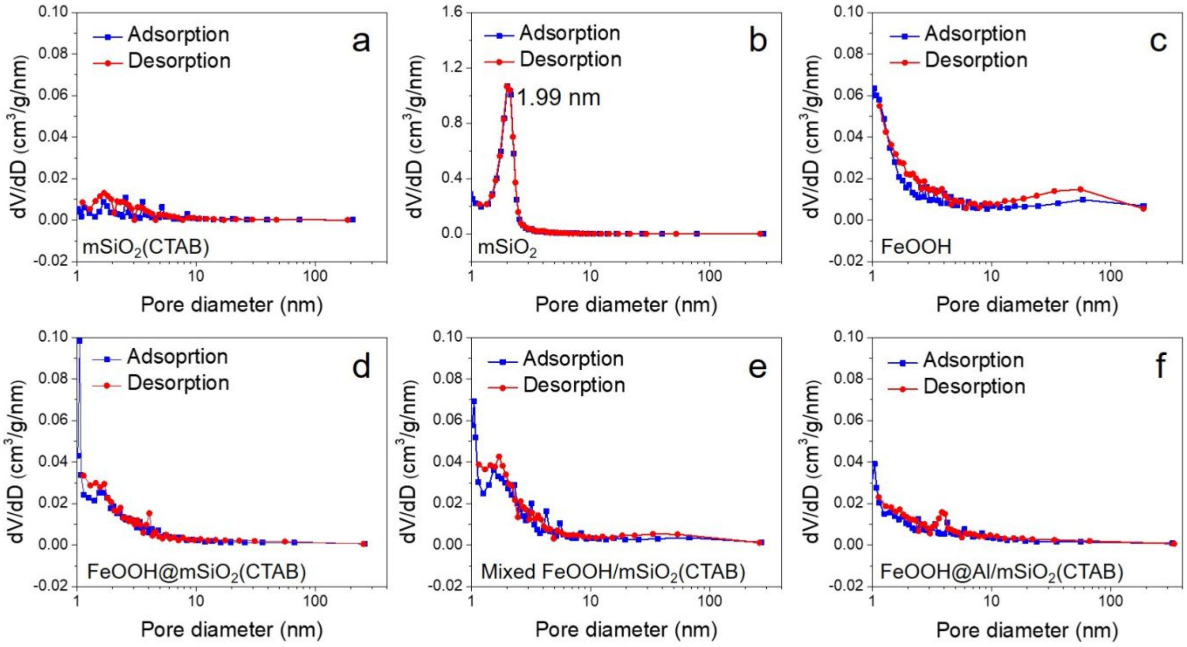

Figure S2. Pore size distribution curves of $mSiO_2$(CTAB) (a) before and (b) after calcination; (c) FeOOH nanosheets; (d) FeOOH@$mSiO_2$(CTAB); (e) Mixed FeOOH/$mSiO_2$(CTAB) and (f) FeOOH@Al/$mSiO_2$(CTAB).

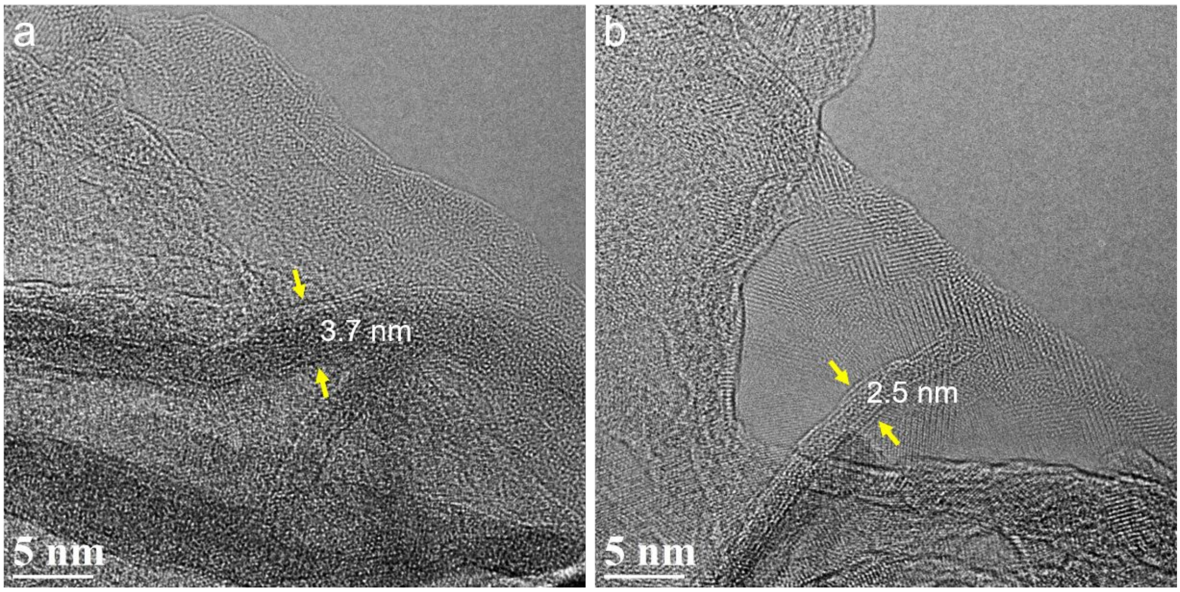

Figure S3. (a,b) HRTEM images of FeOOH nanosheets. The thickness of FeOOH nanosheets is measured as 2-4 nm.

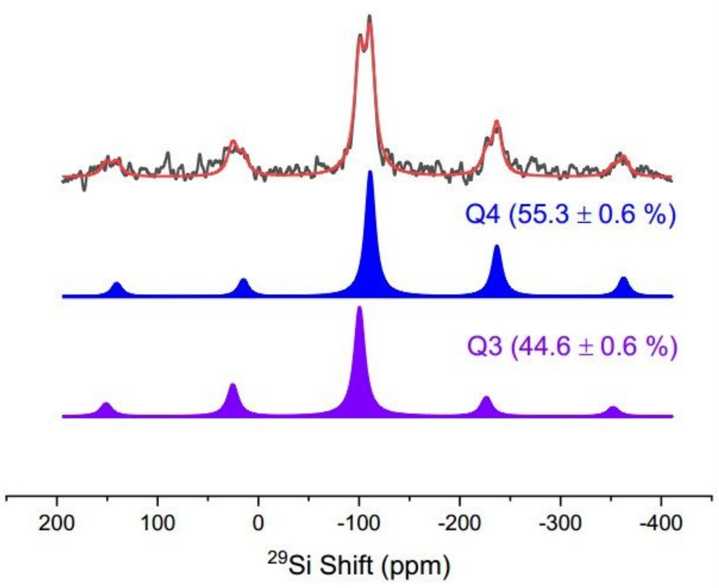

Figure S4. ^{29}Si MAS NMR spectrum of the coated sample without Al dopant.

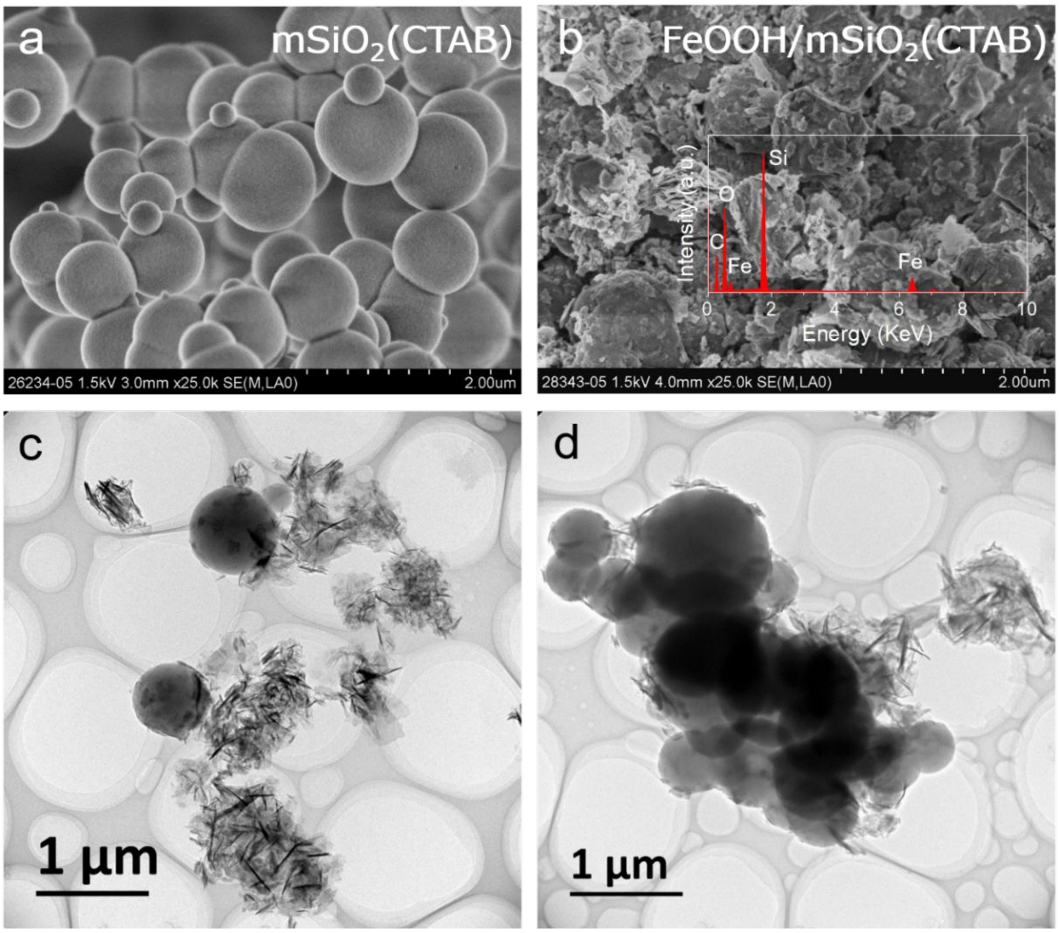

Figure S5. SEM images of (a) $mSiO_2$(CTAB) support and (b) mixed FeOOH/$mSiO_2$(CTAB); (c,d) TEM images of mixed FeOOH/$mSiO_2$(CTAB). Inset of (b) shows the corresponding EDX.

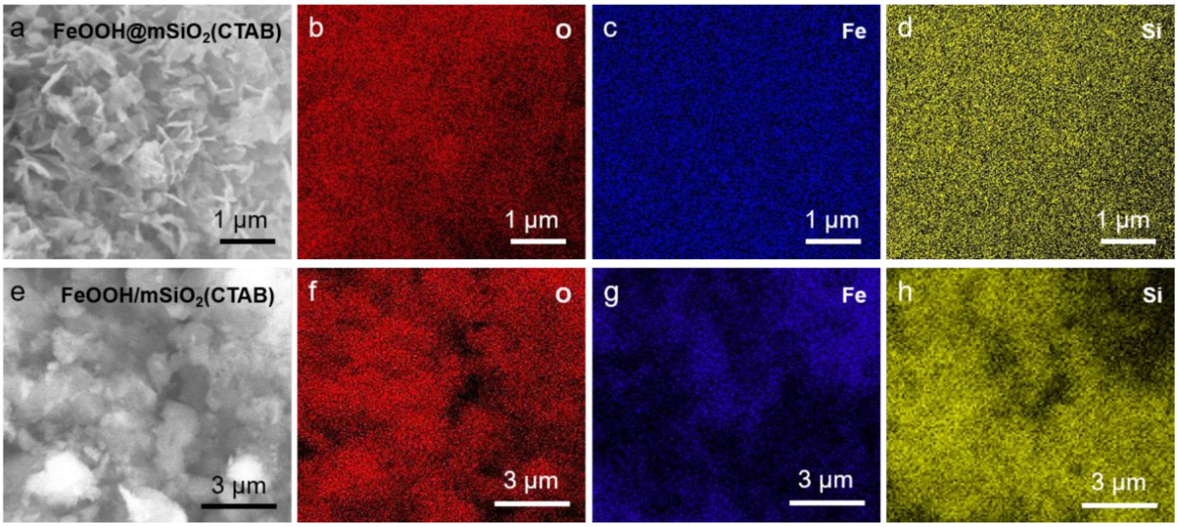

Figure S6. Low-magnification SEM images of (a) FeOOH@mSiO_2(CTAB) and (e) FeOOH/mSiO_2(CTAB); (b-d, f-h) the corresponding elemental maps of O, Fe and Si.

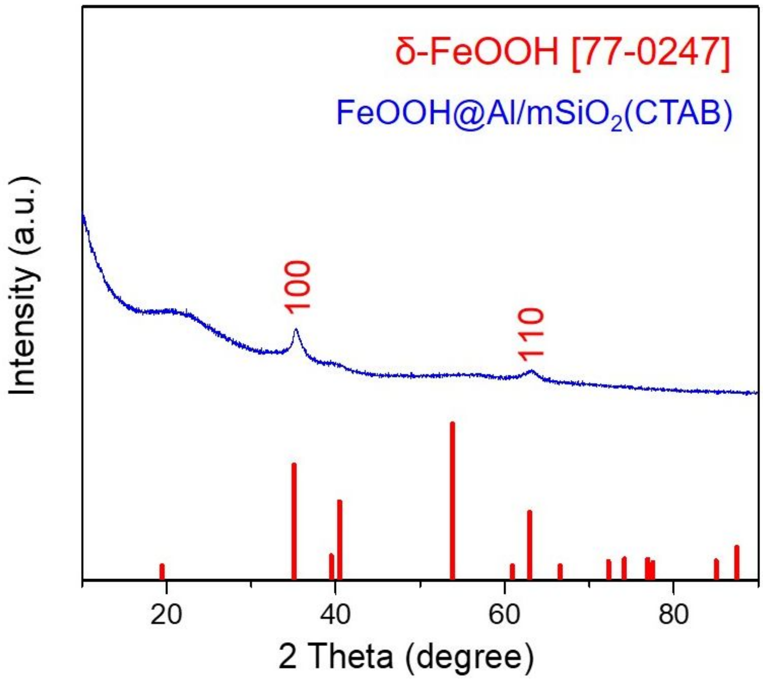

Figure S7. XRD pattern of the coated sample with Al dopant.

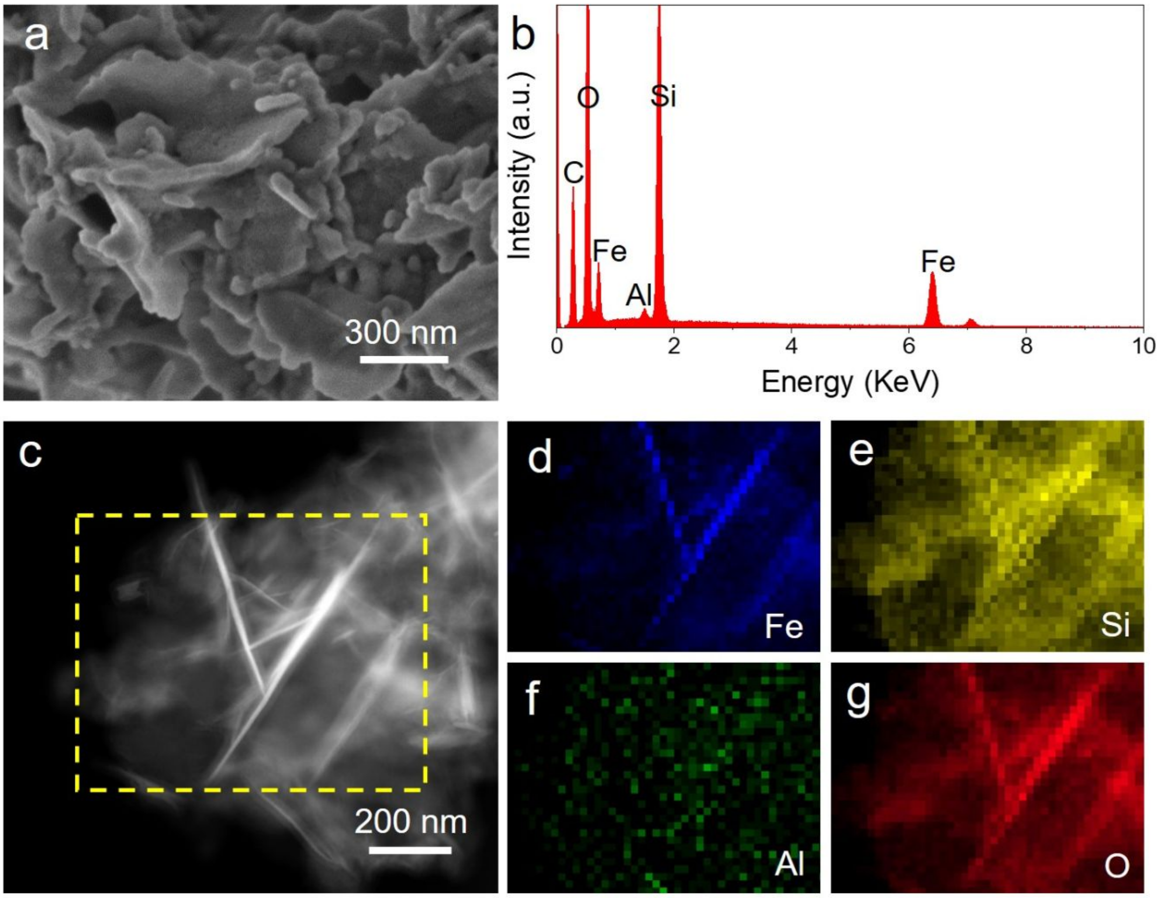

Figure S8. (a) SEM image, (c) HAADF-STEM image and (b, d-g) the corresponding elemental analysis of the coated sample with Al dopant.

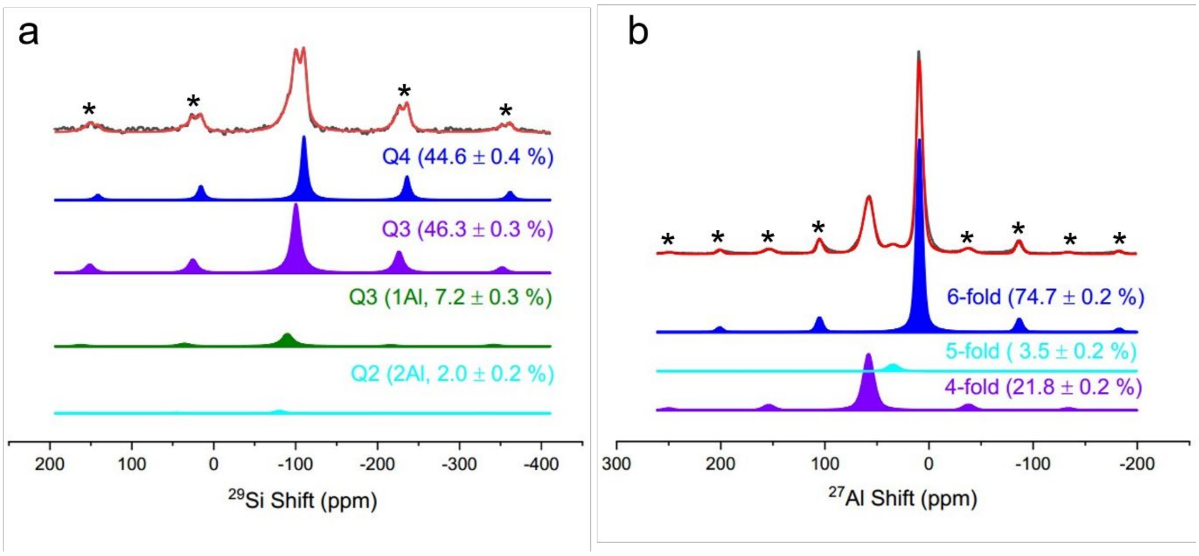

Figure S9. (a) ^{29}Si and (b) ^{27}Al MAS NMR spectra of the coated sample with Al dopant. Spinning sidebands are marked by asterisks.

The width of the MAS sideband pattern is a sensitive probe of the mean distance of the coating from the surface.[S1] Since the diamagnetic coating and the magnetic nanosheet form different phases, the chemical shift distribution is nucleus independent and the sideband patterns of ^{27}Al and for ^{29}Si can be directly compared. The considerably lower width of the sideband pattern for ^{27}Al is, therefore, a direct indication that the Al sites are predominantly located towards the surface of the coating, away from the nanosheets.

The linewidth of the central line in the ^{27}Al MAS spectrum is an additional, although somewhat less direct indicator for the position of the Al. The 4-fold ^{27}Al signal has a full-width-at-half-maximum (FWHM) of 2.6 kHz, while the signal from 6-fold coordinated Al has a FWHM of 1.5 kHz. This indicates that the 4-fold signal is caused by ^{27}Al in a more distorted environment than the 6-fold ^{27}Al, which may be caused by a predominant surface position of the 4-fold Al, while the 6-fold Al signal may be caused by subsurface Al.

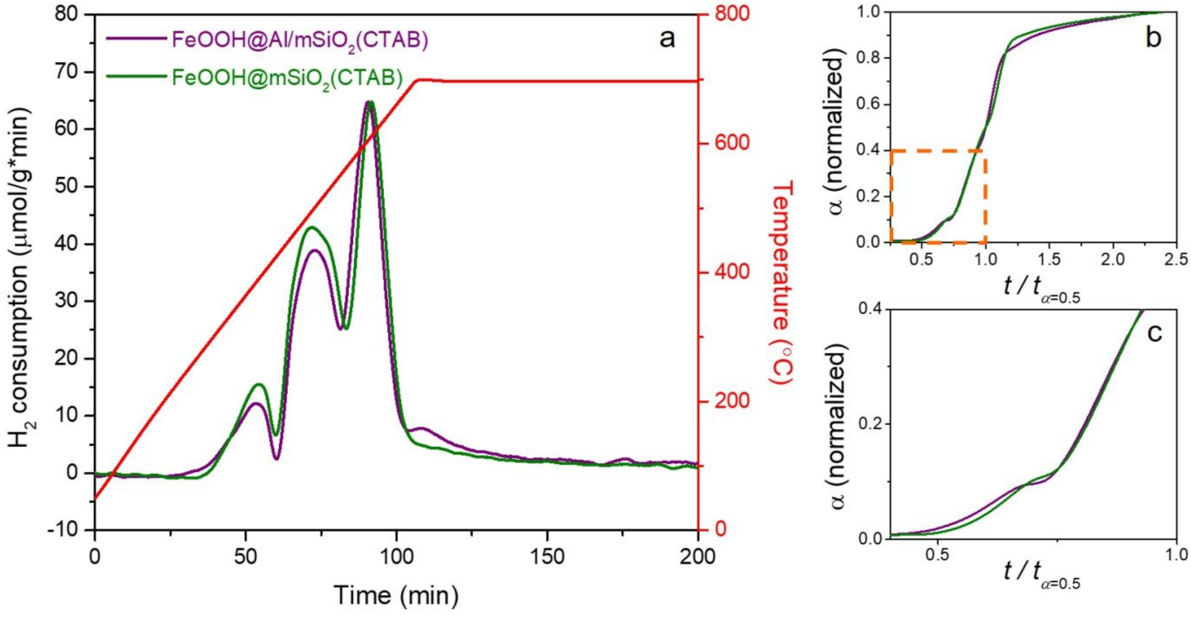

Figure S10. (a) H_2-TPR and (b,c) integrated TPR curves to time-fractions ($t/t_{\alpha=0.5}$) of the coated samples with (purple) and without (green) Al doping. Conditions: 5%H_2/Ar, 80 mL min^{-1}, 6 °C min^{-1}, 700 °C, 90 min holding time.

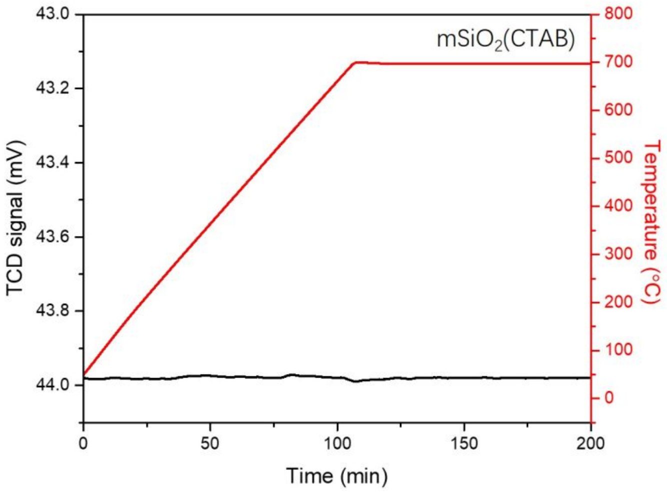

Figure S11. TPR profile of $mSiO_2$(CTAB), showing no obvious presence of H_2 consumption.

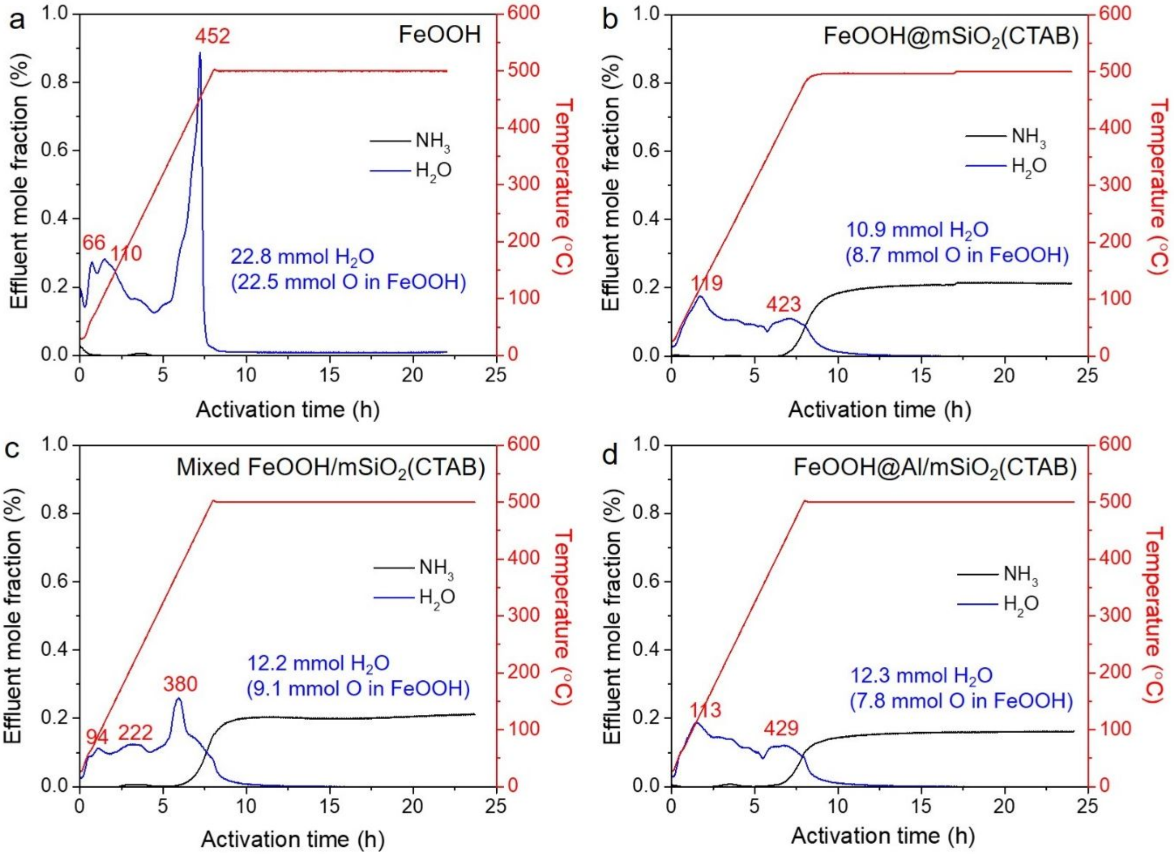

Figure S12. Time course of NH_3 and H_2O evolution during *in situ* activation. Activation conditions: 75%H_2/N_2, 440 NmL min^{-1}, 1 °C min^{-1}, 500 °C, 14-16 h holding time.

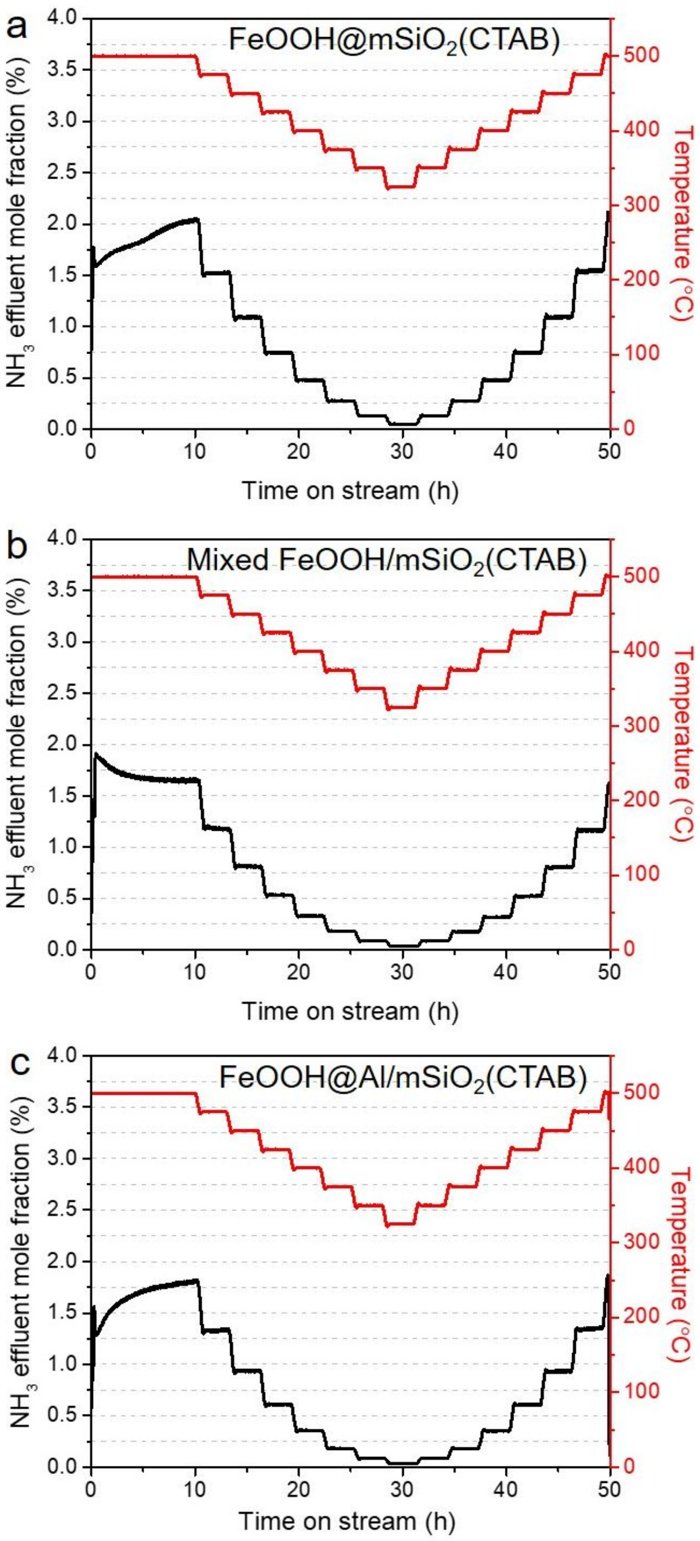

Figure S13. Ammonia effluent mole fraction at different temperatures. All temperatures were kept constant for 155 min.

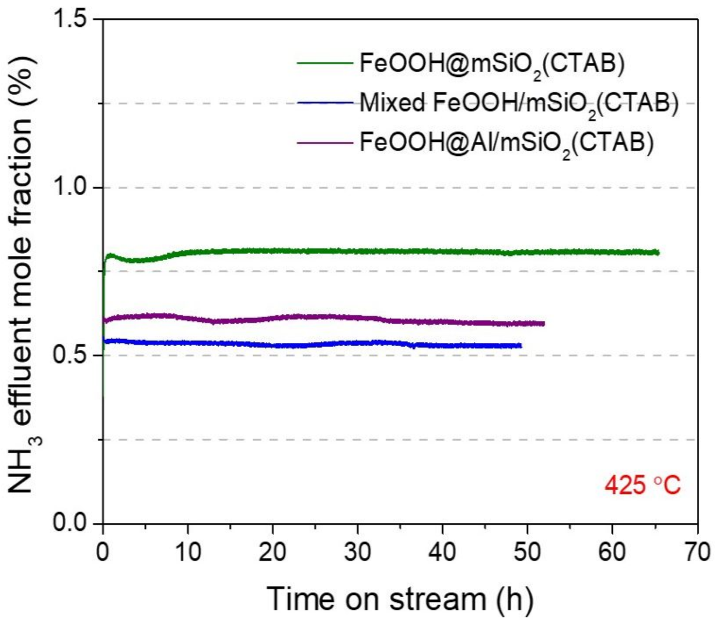

Figure S14. Stability tests on encapsulated and supported catalysts.

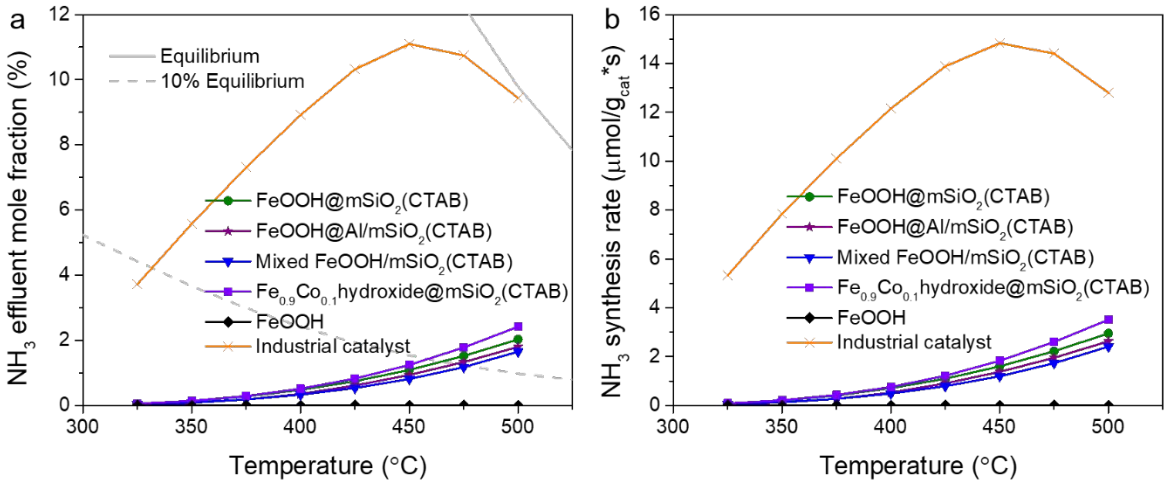

Figure S15. (a) Ammonia effluent mole fraction obtained in steady state at different temperatures under 90 bar; (b) Rate of ammonia synthesis normalized by catalyst mass. The apparent activation energy of the Co-containing sample is 86.0 kJ mol^{-1}.

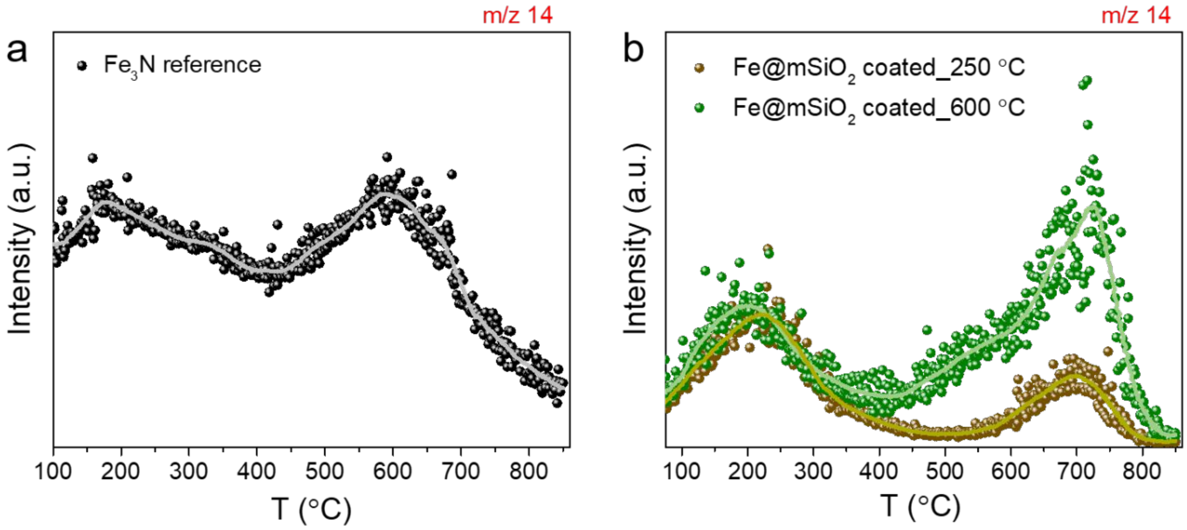

Figure S16. TDS spectra of (a) Fe_3N reference and (b) the $mSiO_2$ coated catalyst treated by 75%H_2/N_2 at 250 °C and 600 °C.

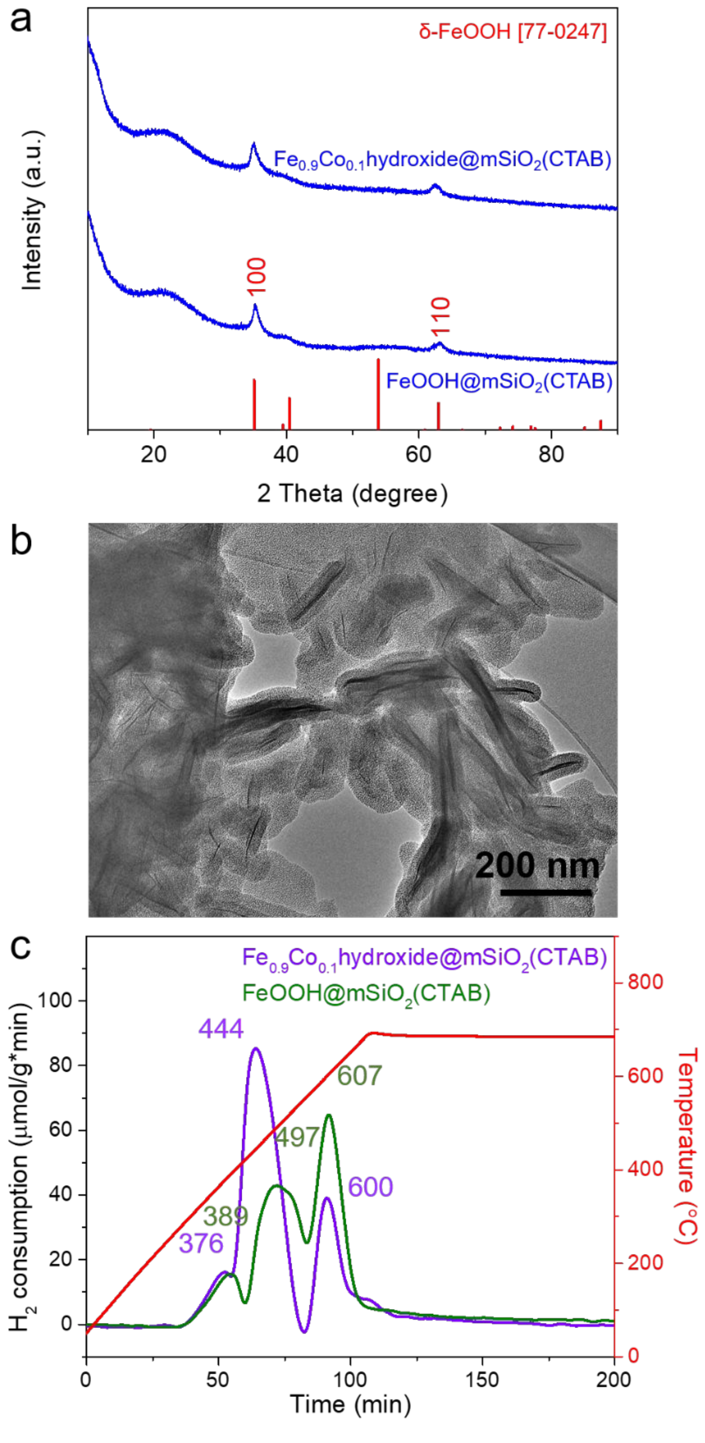

Figure S17. (a) XRD pattern, (b) TEM image and (c) H_2-TPR of $Fe_{0.9}Co_{0.1}$hydroxide@mSiO_2(CTAB). The metal (Fe+Co) loading amount in its reduced state is 35.6 %. The molar ratio of Fe:Co is 8.8.

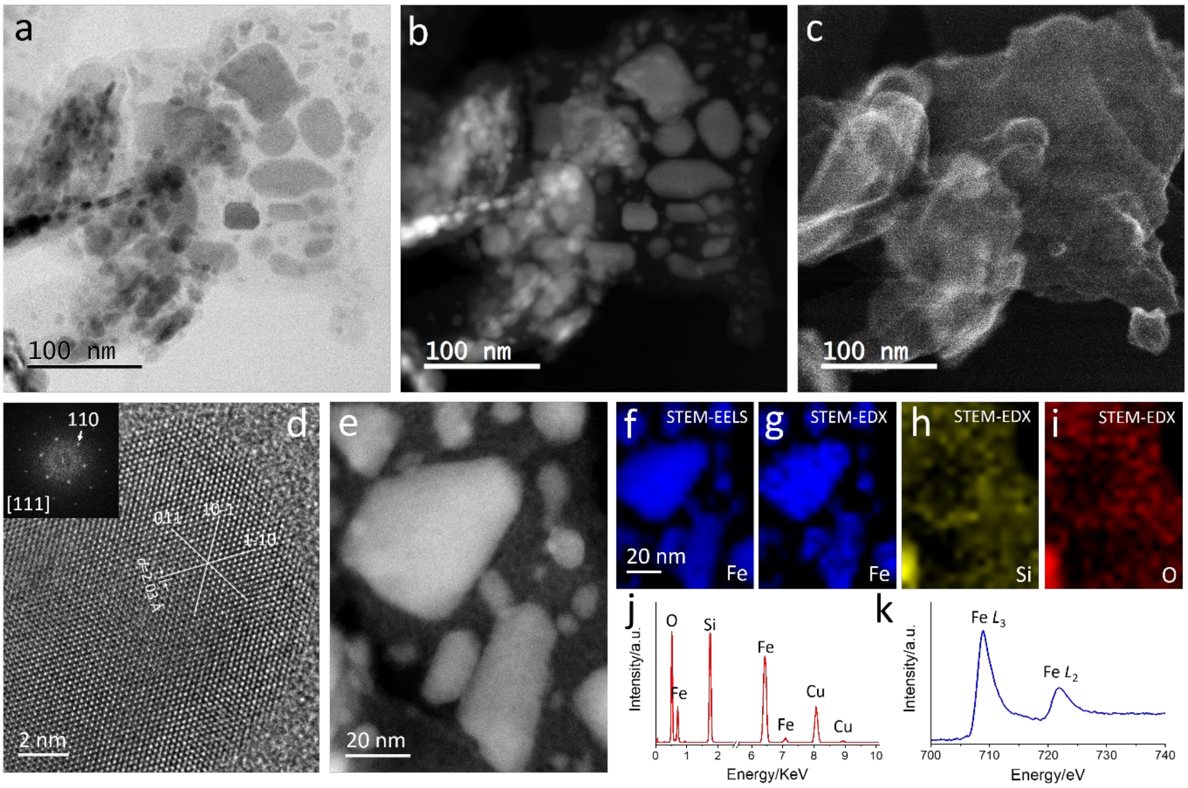

Figure S18. (a) ABF-STEM image, (b) HAADF-STEM image and (c) SE image of spent encapsulated catalyst; (d) HRTEM images of plane view of catalyst, revealing the [111] orientated Fe nanoplate; (e) HAADF-STEM image and (f-i) elemental analysis of coated catalysts; (j) EDX spectrum and (k) EELS spectrum. Note: there was no air contact during the sample transfer from the reaction tube into the chamber of TEM.

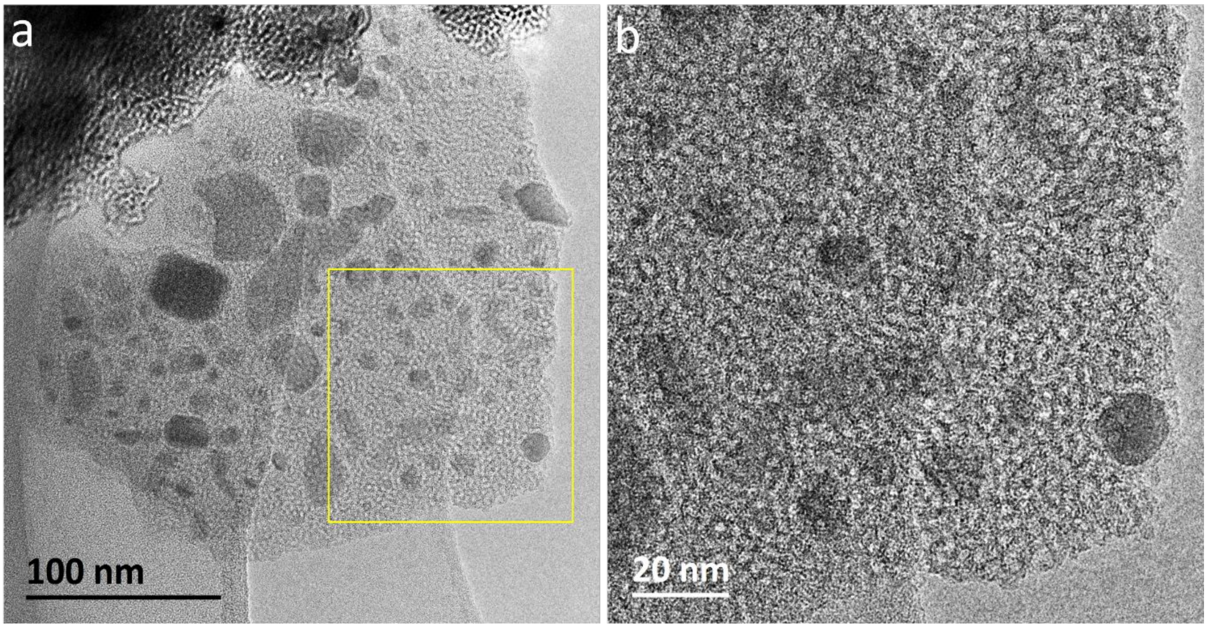

Figure S19. (a,b) TEM images of spent encapsulated catalyst, showing clearly the presence of mesopores in the capping SiO_2 layers.

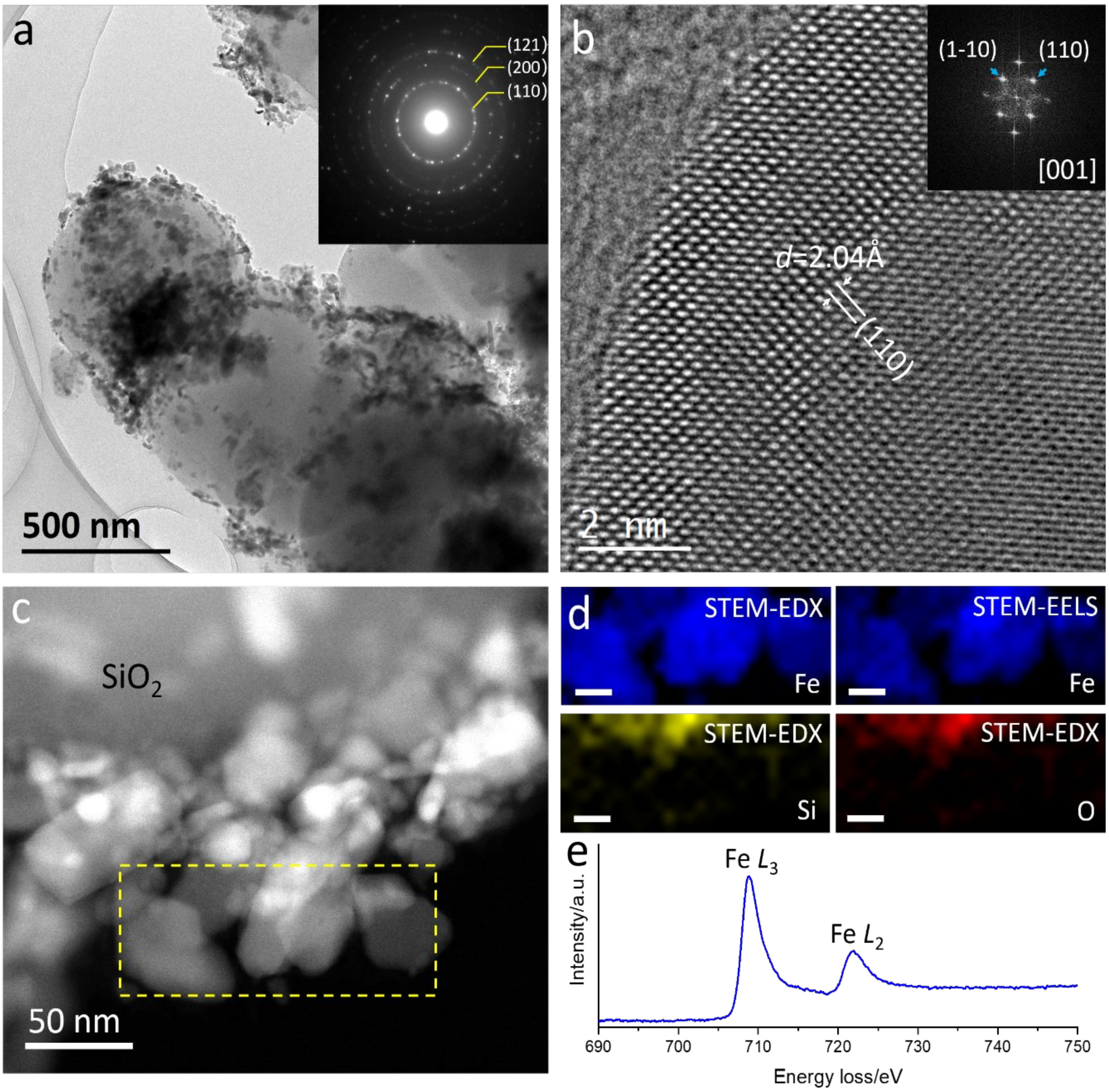

Figure S20. (a) TEM and (b) HRTEM images of supported Fe catalyst after catalytic reaction; Inset of (b) shows the SAED pattern of the sample; (c) HAADF-STEM image and (d) the corresponding STEM-EDX elemental maps of Fe, Si and O as well as STEM-EELS map of Fe; (e) EELS spectrum of Fe; Scale bar in (d) is 20 nm.

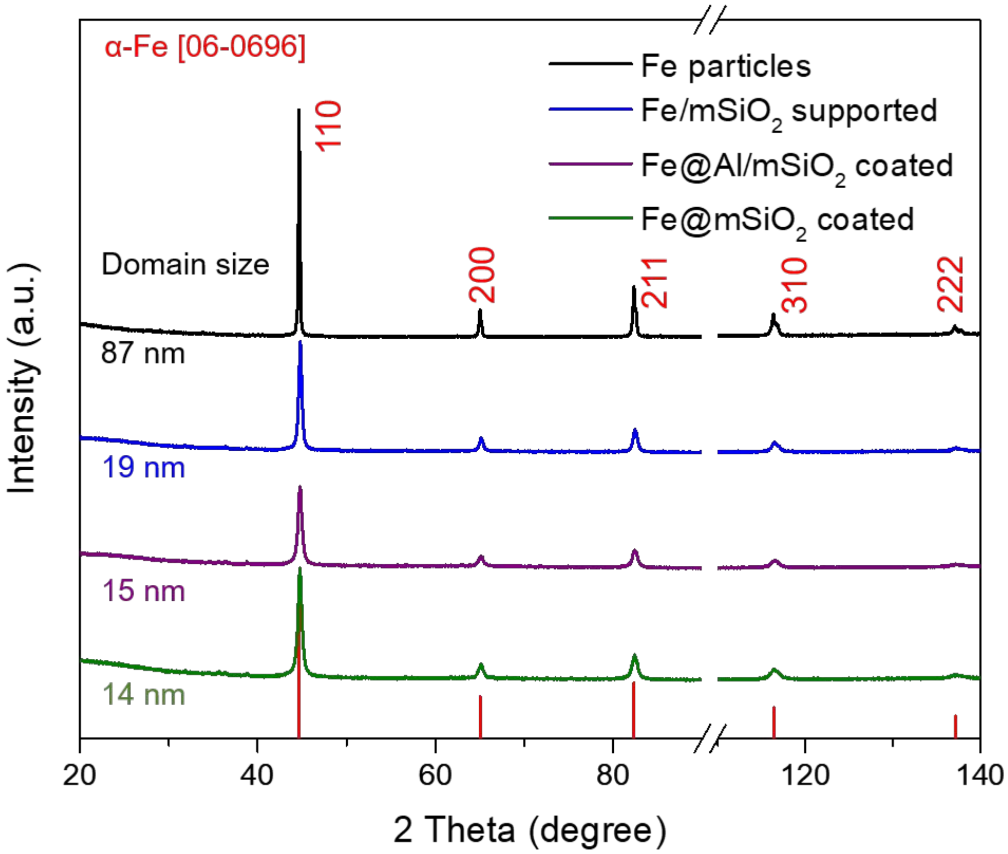

Figure S21. XRD patterns of the catalysts after NH_3 synthesis and the corresponding Fe domain sizes.

Discussion of TEM (Figure S18d) and XRD (Figure S21) data: Our TEM characterization reveals that in the coated catalysts, the (111) terminated 2D Fe plates (top/bottom) are more frequently observed than the (100) or (110) terminated ones (see Figure S18d and Figure 5c,d). This is supported further by analysis of XRD. We find that the intensity ratio of (110)/(222) increases following the order of Fe@Al/$mSiO_2$ coated (7.4) < Fe@$mSiO_2$ coated (7.5) < Fe/$mSiO_2$ supported (9.0) < Fe particles (11.9). This indicates an increased (111) periodicity of Fe particles in above-mentioned samples. The coated samples generally show smaller (110)/(222) ratios, which can be explained by the preferential orientation and thin thickness of 2D Fe plates along the [111] direction. Thus, the coated catalysts are likely to have a higher fraction of (111) surface exposed compared to the supported catalyst.

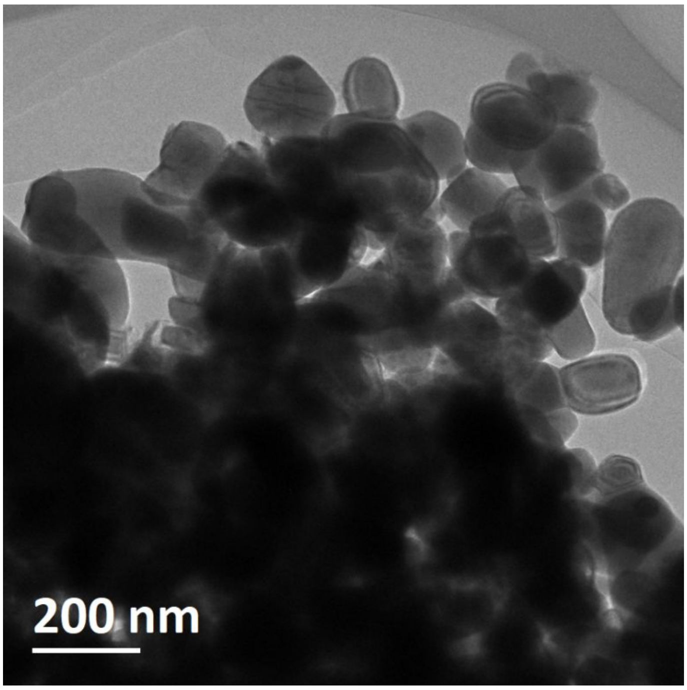

Figure S22. TEM images of the unsupported Fe catalyst after catalytic reaction.

REFERENCES

[S1] E. M. Levin,S. L. Bud'ko. Bulk magnetization and 1H NMR spectra of magnetically heterogeneous model systems. J. Magn. Magn. Mater. 2011, 323, 2355-2361.
